# RNA-Seq analysis of resistant and susceptible sub-tropical maize lines reveals a role for kauralexins in resistance to grey leaf spot disease, caused by *Cercospora zeina*

**DOI:** 10.1186/s12870-017-1137-9

**Published:** 2017-11-13

**Authors:** Jacqueline Meyer, Dave K. Berger, Shawn A. Christensen, Shane L. Murray

**Affiliations:** 1 Department of Plant and Soil Sciences, Forestry and Agricultural Biotechnology Institute (FABI), University of Pretoria, P/Bag X20, Hatfield, Gauteng 0028 South Africa; 2 Center for Medical, Agricultural, and Veterinary Entomology, United States Department of Agriculture, Agricultural Research Service, Chemistry Research Unit, Gainesville, Florida, 32608 USA; 3 Department of Molecular and Cell Biology, University of Cape Town, Private Bag, Rondebosch, Cape Town, 7701 South Africa; 4 Centre for Proteomic and Genomic Research, Upper Level, St Peter’s Mall, Cnr Anzio and Main Road, Observatory, Cape Town, 7925 South Africa

**Keywords:** Grey leaf spot, *Cercospora zeina*, *Zea mays*, Phytoalexin, Kauralexin, RNA-Seq

## Abstract

**Background:**

*Cercospora zeina* is a foliar pathogen responsible for maize grey leaf spot in southern Africa that negatively impacts maize production. Plants use a variety of chemical and structural mechanisms to defend themselves against invading pathogens such as *C. zeina*, including the production of secondary metabolites with antimicrobial properties. In maize, a variety of biotic and abiotic stressors induce the accumulation of the terpenoid phytoalexins, zealexins and kauralexins.

**Results:**

*C. zeina*-susceptible line displayed pervasive rectangular grey leaf spot lesions, running parallel with the leaf veins in contrast to *C. zeina*-resistant line that had restricted disease symptoms. Analysis of the transcriptome of both lines indicated that genes involved in primary and secondary metabolism were up-regualted, and although different pathways were prioritized in each line, production of terpenoid compounds were common to both. Targeted phytoalexin analysis revealed that *C. zeina-*inoculated leaves accumulated zealexins and kauralexins. The resistant line shows a propensity toward accumulation of the kauralexin B series metabolites in response to infection, which contrasts with the susceptible line that preferentially accumulates the kauralexin A series. Kauralexin accumulation was correlated to expression of the kauralexin biosynthetic gene, *ZmAn2* and a candidate biosynthetic gene, *ZmKSL2*. We report the expression of a putative copalyl diphosphate synthase gene that is induced by *C. zeina* in the resistant line exclusively.

**Discussion:**

This study shows that zealexins and kauralexins, and expression of their biosynthetic genes, are induced by *C. zeina* in both resistant and susceptible germplasm adapted to the southern African climate. The data presented here indicates that different forms of kauralexins accumulate in the resistant and susceptible maize lines in response to *C. zeina*, with the accumulation of kauralexin B compounds in a resistant maize line and kauralexin A compounds accumulating in the susceptible line.

**Electronic supplementary material:**

The online version of this article (10.1186/s12870-017-1137-9) contains supplementary material, which is available to authorized users.

## Background

Cereal crops such as maize (*Zea mays*), wheat *(Triticum aestivum*), and rice (*Oryza sativa*) are essential components of consumer diets throughout the world. In 2013, Africa produced over 55 million tonnes of cereal products (www.fao.org/worldfoodsituation/csdb/en/). Fungal infections are detrimental to maize crops, severely limiting crop yield, quality and productivity [1]. Grey leaf spot (GLS) is a foliar disease prevalent in South Africa, especially in Kwazulu Natal, where hot and humid climate conditions are favourable for the development of the disease [1]. A genetically distinct *Cercospora* sp. endemic to the eastern corn belt in USA, China and sub-Saharan Africa was identified as the causal agent for GLS in southern Africa and classified as *C. zeina,* the focus of this study [2–4]. Fungicide control is expensive and not necessarily effective and *C. zeina* is rampant in southern Africa [1], severely reducing grain yields, with losses of up to 60% reported [5]. It is believed that government incentives to reduce tilling practices contributed to an increase in disease prevalence. In so doing, *C. zeina* spores are allowed to remain on dead leaf material from previous harvests, readily infecting subsequent crops [1].

Plants defend themselves through a variety of mechanisms, including the production of secondary metabolites [6]. Several non-volatile terpenoids are known to act as phytoalexins in rice and maize [7, 8]. Phytoalexins are antimicrobial molecules synthesised de novo after pathogen attack and act to inhibit the growth of the invading pathogen [9]. In rice, the universal diterpenoid precursor, geranyl-geranyl diphosphate (GGPP), is converted to two stereochemically differentiated isomers, *ent-* and *syn-*copalyl diphosphate (CDP). This conversion entails a cyclization that is catalyzed by two discrete copalyl diphosphate synthases (CPS), *ent*- and *syn*-CPS [10]. Gibberellin (GA), a ubiquitous, diterpenoid plant hormone responsible for growth and development, and diterpenoid phytoalexins share a common biosynthetic step catalyzed by *ent-*CPS in rice [10]. *Syn*-CPS exclusively produces diterpenoid phytoalexins [10]. Subsequent synthesis steps in rice GA synthesis involve the enzyme *ent*-kaurene synthase (KS) which yields *ent*-kaurene, followed by repeated oxidation steps by means of cytochrome P450 monooxygenases until GA is produced [11]. Similarily, diterpenoid phytoalexins are produced by diterpene synthases, termed kaurene synthase-like (KSL) enzymes, so named for their apparent homology to *ent*-KS. In rice there are four structurally distinct diterpenoid phytoalexins; phytocassanes A-E, oryzalexins A-F, momilactones A and B, and oryzalexin S [8], produced, respectively from the kaurene-like precursors *ent*-cassadiene, *ent*-sandaracopimaradiene, *syn*-pimaradiene and stemarene [8, 12]. Whilst phytocassanes and oryzalexins stem from the primary building block *ent*-CDP by means of KSL enzymes, momilactones and oryzalexin S stem from *syn*-CDP [13, 14]. The remaining reactions resulting in rice phytoalexins are catalyzed by cytochrome p450 family members [15–17].

**Fig. 1:**
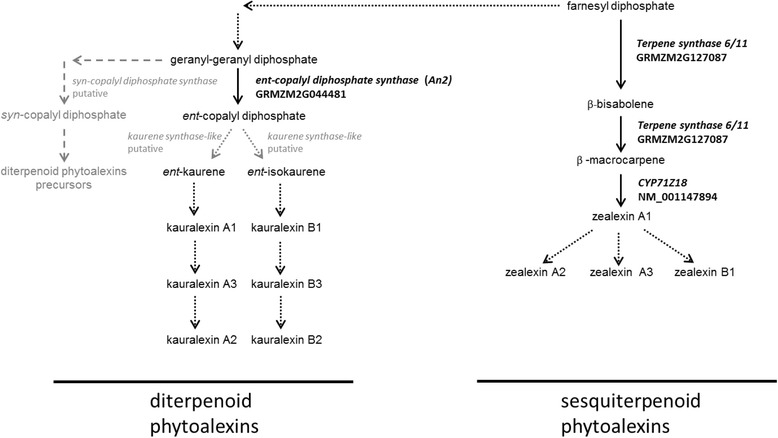
Biosynthetic pathways leading to kauralexin and zealexin compounds in maize. Enzymes indicated in bold and reactions represented by solid arrows have been established empirically, while dashed arrows indicate gaps in our knowledge. Enzymes and reactions in greyscale have been predicted in silico. Pathway adapted from Plant Metabolic Network (PMN), on www.plantcyc.org, August 2015. The general pathway structure is proposed based on the order of enzymatic conversions observed in other terpenoid biosynthetic pathways, such as in rice [12]

Similar to rice, maize kauralexins are diterpenoid phytoalexins that are fungal-induced and occur as a result of the action of the *ent*-CPS, ZmAn2 [7, 18]. The *ZmAn2* gene product shares 60% amino acid sequence identity with the maize ZmAn1 enzyme that functions in GA biosynthesis [18]. ZmAn2 catalyzes the cyclization of GGPP to *ent-*CDP (Fig. 1). Thereafter, *ent-*CDP is transformed to *ent*-kaurene and *ent*-isokaurene - most likely through the action of two distinct kaurene synthase-like enzymes - which yield kauralexin A and B series of compounds, respectively (Fig. 1) [19]. The plausible pathway structure in maize is envisioned based on the order of enzymatic conversions observed in other terpenoid biosynthetic pathways, such as the rice diterpenoid phytoalexin biosynthetic pathway [12, 20]. Unlike rice, maize diterpenoid phytoalexins resulting from *syn*-CDP as an intermediate have not been discovered to date (Fig. 1).

The remaining reactions in the kauralexin pathway require oxygen, and are predicted to be catalyzed by cytochrome p450 monooxygenase enzymes as reported in rice, although no empirical evidence exists as of yet [11]. The p450 enzymes are expected to catalyze the conversion of specific carbon atoms to carboxylic acids via the formation of alcohols and their conversion to aldehydes (forming kauralexin A1 and kauralexin B1). Similarly, using a different carbon atom, the aldehydes, kauralexin A3 and kauralexin B3, will be formed and subsequently the dicarboxylates (kauralexin A2, kauralexin B2) (Fig. 1). Diterpenoid kauralexins were shown to be induced in maize roots by drought, *Fusarium verticillioides* and *Phytophthora cinnamomi*, in maize leaves by *C. zeina* and in maize stems by *F. graminearum* and *Rhizopus microsporus* infection [7, 18, 21–23].

Besides diterpenoid kauralexins, maize also accumulates sesquiterpenoid zealexins as a general defense response to a fungal attack [24, 25]. These compounds are structurally related to β-macrocarpene and two homologous terpene synthases, TPS6 and TPS11, are known to catalyze the production of β-macrocarpene from the precursor farnesyl diphosphate via β-bisabolene [26] (Fig. 1). The sesquiterpenoid biosynthetic genes, *Tps6* and *Tps11*, are induced and correlate to zealexin accumulation in response to a variety of biotic stressors such as *F. graminearum* infection, *Ostrinia nubilalis* herbivory, *Cochliobolus heterostrophus* and *U. maydis* [7, 24, 25, 27, 28]. Furthermore virus-induced gene silencing of *Tps6* and *Tps11* resulted in plants with an augmented susceptibility to *U. maydis* [29]. Zealexin and kauralexin accumulation appears to be co-regulated [25]. It has been reported that both compounds, as well as the expression of their biosynthetic genes, increase when spores of *F. graminearum* or *A. flavus* are applied in increasing inoculum [25].

Although fungal induced phytoalexins have been studied widely in maize roots, stems and ears, much less is known concerning fungal induced phytoalexins in leaves. We recently showed that kauralexin biosynthetic genes are co-regulated in a susceptible response to *C. zeina* [21] but also induced in a moderately resistant maize line in response to *C. zeina* (B Crampton, pers. comm.). This led us to question if different kauralexin biosynthetic gene paralogs are involved in a susceptible and resistant response in a recombinant inbred population [21] to this pathogen. In this study, we aimed to use RNA-sequencing to identify paralogues of known secondary metabolite biosynthetic genes as well as other potential molecular mechanisms of susceptibility and resistance to GLS. Furthermore, by using a priori knowledge of the genomic positions of quantitative trait loci (QTL) mapping for GLS disease severity in a CML444 X SC Malawi RIL population [21], we could determine if there was a putative genetic mechanism that underlying these defense responses. Finally, we aimed to characterize the phytoalexin accumulation that occurs in maize leaves in response to *C. zeina* infection by targeted metabolomic screening for zealexin and kauralexin compounds.

## Results

### Identification of RILs with extreme resistance and susceptibility to *C. zeina*

**Fig. 2:**
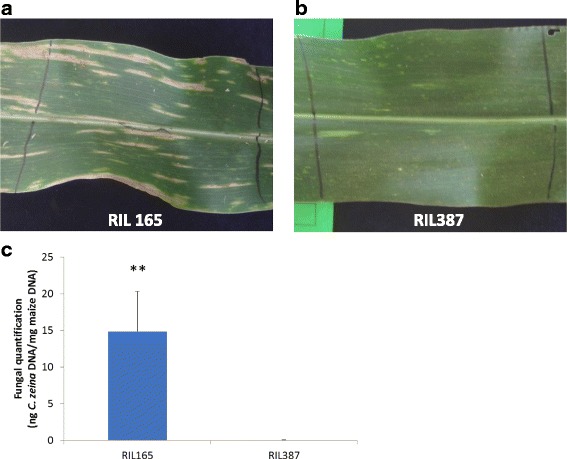
Comparison of disease lesions in *C. zeina*-susceptible and *C. zeina*-resistant lines. Leaf material was harvested at 103 dap. **a** *C. zeina*-susceptible line (RIL165) displayed mature grey leaf spot lesions, tan, rectangular in shape and running parallel with the leaf veins. **b** *C. zeina*-resistant line (RIL387) showed limited disease symptoms. **c** *In planta* fungal quantification indicated that leaf material from RIL165 contained significantly higher amounts of fungal DNA than leaves from RIL387. A qPCR assay could determine ng *Cercospora zeina (Cz)* DNA per mg maize DNA [31]. Input DNA was extracted from leaf material (average quantity, *n* = 3 ± SEM; unpaired T-test, **P < 0.01)

During a previous study, field trials of maize recombinant inbred lines (RIL) derived from a cross between CML444 and SC Malawi (F7:S6) were conducted and eight quantitative trait loci (QTL) for GLS disease severity were identified [21]. During the field trials, two RILs with extreme resistance or susceptibility to GLS were identified. RIL387 and RIL165 had different alleles at four of the eight QTL previously identified for severity of GLS disease (Additional file 1) [21], with only RIL387 carrying the resistance allele for three QTL (QTL6, QTL9b and QTL10) and RIL165 only exhibiting the resistance allele for QTL9a. Both lines carried the resistance allele for the remaining four QTL (QTL3a, QTL3b, QTL4 and QTL5; Additional file 1). At 103 days after planting (dap), mature GLS disease symptoms were present and easily distinguishable. Disease severity could be rated effectively [30]. The *C. zeina*-susceptible line (RIL165) displayed mature grey leaf spot lesions (tan, rectangular and running parallel with the leaf veins) and obtained a disease severity score of 7.5 (numerous lesions on upper leaves with majority of lower leaves dead [30] (Fig. 2a). The *C. zeina*-resistant line (RIL387) exhibited a disease severity score of 1.5 (limited disease symptoms; Fig. 2b). To identify potential molecular mechanisms of resistance, leaf material was harvested from these two lines for transcriptome (RNA) sequencing. *In planta* fungal quantification of harvested leaf material (103 dap) [31] indicated that RIL165 leaves contained significantly higher levels of fungal DNA than leaves from RIL387 (Fig. 2c).

### Transcriptome profiling to identify differentially expressed (DE) genes between *C. zeina* resistant and susceptible maize lines

RNA isolated from resistant (RIL387) and susceptible (RIL165) maize leaves were subjected to RNA-Seq. A summary of the analysis pipeline is depicted in Additional file 2. Sequence reads were mapped to v2 of the B73 reference genome (5b.60 annotation) using TopHat and implementing Bowtie2. The number of reads obtained per sample after Illumina quality filtering ranged from 59.3–78.8 million, and 75–83% of reads per library mapped to the reference genome (Table 1). Thereafter Cufflinks was used to calculate transcript abundance, reported as fragments per kilobase pair of exon model per million fragments mapped (FPKM).

**Table 1:** Read mapping, alignment and subsequent expression summary for RIL165 and RIL387

Sample^a^	Number of quality filtered reads	Number of mapped reads^b^	Number of paired alignments	Maximum FPKM^c^	Number of expressed genes
RIL165_1	70,769,770	83%	27,884,716	5761	22,558
RIL165_2	62,302,258	75%	21,984,219	6882	22,895
RIL165_3	78,883,890	76%	28,417,469	4868	22,773
RIL387_1	62,399,266	80%	23,331,358	6755	22,633
RIL387_2	63,798,694	78%	23,458,704	8659	22,575
RIL387_3	59,304,854	80%	22,353,073	6349	22,641

^a^Samples are numbered per biological replicate

^b^Sequence reads for each biological replicate were mapped to v2 of the B73 reference genome using TopHat v2.0.9 [103]

^c^Cufflinks v2.0.2 was used to calculate transcript abundance which is reported as fragments per kilobase pair of exon model per million fragments mapped (FPKM) [33]

We detected between 22,000 and 23,000 protein coding genes per biological replicate (Table 1). In all three biological replicates of each genotype, the maximum FPKM values obtained were 6882 in RIL165 and 8659 in RIL387 while minimum FPKM values were zero (Table 1). A gene was considered expressed if the lower value of the 95% confidence interval was greater than zero [32].

Differential expression analysis was conducted using Cuffdiff [33, 34]. The False Discovery Rate (FDR) adjusted *P*-value threshold was set to 0.05 and revealed 5648 genes differentially expressed between RIL165 and RIL387 (Additional file 3). Differentially expressed genes were further shortlisted by selecting genes with a Log_2_fold change (Log_2_FC) ≥1 or ≤ − 1 for further analysis (Additional file 3). Of these genes, 328 mapped to the chromosomal position of the eight disease severity QTL previously identified [21], and 151 DE genes mapped to QTLs with different alleles in RIL387 and RIL165, viz. QTL6 (23 genes); QTL9a (99 genes); QTL9b (13 genes) and QTL10 (16 genes) (Additional file 1 and Additional file 3). To dissect responses specific to RIL165 and RIL387, 1517 genes with a Log_2_FC ≥ 1 were defined as having higher expression in RIL387 compared to RIL165 and 2577 genes with Log_2_FC ≤ −1 as having increased expression in RIL165 compared to RIL387. The RNA-Seq expression profiles of DE genes were validated by RT-qPCR. Eight genes were selected which had high or low FPKM values. There was a strong consistency between genotype specific RT-qPCR relative quantities and RNA-Seq FPKM values (Additional file 4). Larger differences were observed in RNA-Seq FPKM values between genotypes compared to RT-qPCR relative quantities, indicating a higher dynamic range for RNA-Seq analysis, as previously observed [35].

### Gene ontology enrichment and pathway analysis reveals a complex defence response to *C. zeina*

**Fig. 3:**
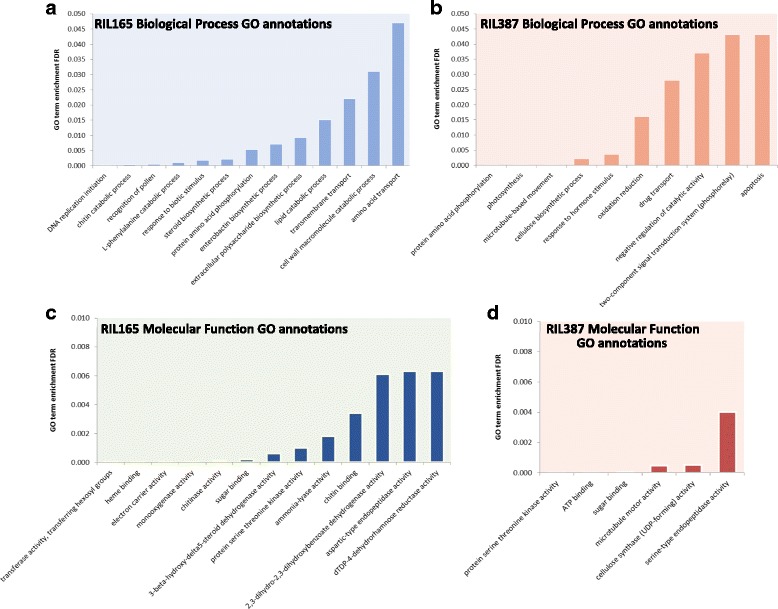
Enriched GO annotations for genes with altered expression in RIL165 and RIL387. Significantly enriched GO terms related to biological processes (**a** and **b**) or molecular functions (**c** and **d**) of the functionally annotated genes with higher expression in RIL165 (*blue bars* in **a** and **c**) or in RIL387 (*red bars* in **b** and **d**). The bars represent the FDR adjusted *p*-value (y-axis), and therefore the terms on the left-hand side of each graph have the most significant enrichment. Only the specialized child terms from the SEA analysis with FDR adjusted *P* value ≤0.05, relating to BP and FDR adjusted P value ≤0.01 relating to MF are shown. A list of all significant GO terms is available in Additional file 5

To determine if DE genes responsive to *C. zeina* infection had any functional connection in defence, GO term singular enrichment analysis (SEA) [36] was carried out on the DE genes (Additional file 5). This resulted in 60 enriched biological process (BP), 61 molecular function (MF) and 5 cellular component (CC) terms for genes with increased expression in *C. zeina-*susceptible RIL165. For highly expressed genes in *C. zeina*-resistant RIL387, 46 BP, 57 MF and 7 CC terms were enriched (Additional file 5). Because of the large number of significant GO terms, for this analysis, only child terms (FDR ≤0.01) relating to BP and MF will be discussed (Fig. 3; Additional file 5). The gene IDs that were associated with each of the enriched GO terms are presented in Additional file 5.

Enriched BP GO terms for genes with higher expression in *C. zeina*-susceptible RIL165 (Log_2_FC ≤ −1; FDR <0.05), included inter alia “L-phenylalanine metabolism” (GO:0006559), “lipid catabolic process” (GO:0016042), “chitin catabolic process” (GO:0006032) and “response to biotic stimulus” (GO:0009607) (Fig. 3a; Additional file 5). This is in line with unique enriched MF GO terms such as “chitinase activity” (GO:0004568) and “chitin binding” (GO:0008061) (Fig. 3b; Additional file 5). The list of DE genes annotated with these GO terms include genes encoding endochitinases (GRMZM2G099454, GRMZM2G062974, GRMZM2G145518); Wound-induced protein WIN1 (GRMZM2G117989); S-norcoclaurine synthase-like (GRMZM2G342033); and four PR proteins (PR1: GRMZM2G112524, GRMZM2G112538, and PR 10: GRMZM2G112488, GRMZM2G075283) (Additional file 3). Apart from DE genes annotated with enriched GO terms, we also identified DE genes encoding five Bowman–Birk serine proteinase inhibitors (GRMZM2G075315, GRMZM2G050768, GRMZM2G116520, GRMZM2G055802, GRMZM2G007928), and four putative thaumatin-like PR genes (GRMZM2G006853, GRMZM2G039639 GRMZM2G138896, GRMZM2G036826). Jasmonic acid biosynthetic genes as well as ethylene (Et)-biosynthetic and -responsive genes were likewise present. Putative JA biosynthetic genes included lipoxygenase genes (GRMZM2G109130, GRMZM2G104843, GRMZM5G822593, GRMZM2G102760, GRMZM2G017616,) allene oxide synthases (GRMZM2G376661 GRMZM2G033098, GRMZM2G067225), an allene-oxide cyclase (GRMZM2G077316), whilst putative Et biosynthetic genes included 1-aminocyclopropane-1-carboxylate oxidase (ACO) – (GRMZM2G126732, GRMZM2G332423, GRMZM2G166616), and Et responsive transcription factor genes (GRMZM2G300924, GRMZM2G016434, GRMZM2G087059, GRMZM2G123119, GRMZM2G425798, GRMZM2G129674, GRMZM2G114820, GRMZM2G068967, GRMZM2G055180, GRMZM2G148333, GRMZM2G174917, GRMZM2G168393, GRMZM2G119865, GRMZM2G080516) (Additional file 3). Furthermore, six genes predicted to encode phenylalanine ammonia lyase (PAL) transcripts contributed to the enriched GO term, “L-phenylalanine metabolism” (GO:0006032) in RIL165 (GRMZM2G063917, GRMZM2G170692, GRMZM2G074604, GRMZM2G081582, GRMZM2G334660, GRMZM2G118345) (Additional file 3 and Additional file 5). In addition, transcripts encoding enzymes that are predicted to partake in further flavonoid biosynthesis - 4-coumarate coenzyme-A ligase (GRMZM2G048522, GRMZM2G174732), chalcone synthase (GRMZM2G422750, GRMZM2G151227), chalcone-flavonone isomerase (GRMZM2G175076, GRMZM2G155329), flavanone 3-hydroxylase (GRMZM2G050234), dihydroflavonol-4-reductase (GRMZM5G881887) - were correspondingly increased in RIL165 compared to RIL387 (Additional file 3). Transcripts from four enzymes that are predicted to catalyze reactions in the shikimate pathway were also more abundant in RIL165, particularly genes encoding a putative 3-dehydroquinate dehydratase (GRMZM2G314652), shikimate kinase (GRMZM2G004590), prephenate dehydratase (GRMZM2G125923, GRMZM2G466543, GRMZM2G437912), and aspartate aminotransferase (GRMZM2G094712) (Additional file 3). In summary, *C. zeina*-susceptible RIL165 shows hallmarks of a multi-layered defence response involving PR proteins, JA/Et production and accumulation of flavonoids.

Genotype specific BP GO terms for genes with higher expression in *C. zeina*-resistant RIL387 (Log_2_FC ≥ 1) included inter alia “photosynthesis” (GO:0015979), “response to hormone stimulus” (GO:0009725), “cellulose biosynthetic process” (GO:0030244), “microtubule-based movement” (GO:0007018) and “apoptosis” (GO:0006915) (Fig. 3b; Additional file 5). This is in line with unique enriched MF GO terms such as “microtubule motor activity” (GO:0003777), “cellulose synthase (UDP-forming) activity” (GO:0016760) (Fig. 3d; Additional file 5) and unique enriched cellular compartmentalisation (CC) GO terms “photosystem I” (GO:0009522), “photosystem II” (GO:0009523) and thylakoid (GO:0009579) (Additional file 5). The list of differentially expressed genes annotated with these GO terms contain eight genes encoding putative cellulose synthases (GO:0030244; GRMZM2G027723, GRMZM2G339645, GRMZM2G111642, GRMZM2G112336, GRMZM2G349834, GRMZM2G018241, GRMZM2G025231, and GRMZM2G028353) and seven putative auxin responsive transcription factors (ARF; ARF1, 3, 4, 6, 7, 15, and 20) (GO:0009725; GRMZM2G441325, GRMZM2G017187, GRMZM2G102845, GRMZM2G475882, GRMZM2G475263, GRMZM2G030710 and GRMZM2G078274). Typically, resistant plants block attack by biotrophic or hemibiotrophic pathogens by initiating localized cell death through the hypersensitive response (HR) [37]. Enriched GO terms relating to the hypersensitive response were absent except for “apoptosis” (GO:0006915). Ten genes contributed to this enriched GO term. A MaizeGDB (www.maizegdb.org) search of the gene ID’s revealed that the best *Oryza sativa* or *Arabidopsis thaliana* hit for these genes were BAG domain containing proteins (GRMZM2G029863 and GRMZM2G063162; GRMZM2G079956) or nucleotide-binding leucine-rich-repeat (NB-LRR) and NB-ARC domains-containing proteins (GRMZM2G079082, GRMZM2G051502, GRMZM2G013170, GRMZM2G002656, GRMZM2G403407 and GRMZM2G169584) as well as a putative stripe rust resistance protein Yr10 (GRMZM2G074496). BAG (Bcl-2-associated athanogene) proteins inhibit plant programmed cell death [38] and NB-LRR and NB-ARC domains are signalling motifs present in plant resistance proteins that also regulate cell death [39]. The NB-LRR and NB-ARC domains could play a role in the maize immune signalling response that results in an oxidative burst and a hypersensitive response, thus indicating that pathogen-recognition mechanisms are possibly up-regualted in RIL387, although in our field experiments, RIL387 developed no macroscopic HR lesions (Fig. 2b). In summary, the resistant line appears to be maintaining structural integrity by upregulating various metabolic pathways, including hormone biosynthesis.

Several enriched BP child terms were common among the two genotypes, however, despite having similar enriched GO terms, there was substantial heterogeneity in the gene pool contributing to the enriched GO terms, with no overlap between the genotypes, indicating distinct responses to *C. zeina* infection. For instance, genes annotated with “oxidation reduction” (GO:0055114) included 22 genes highly expressed in RIL387 and functionally annotated as having monooxygenase activity. These included three benzoxazinoid (*BX*) biosynthetic genes, *Bx3* (GRMZM2G167549)*, Bx4* (GRMZM2G172491), and *Bx5* (GRMZM2G063756)*,* known to encode enzymes which catalyze three successive reactions in the BX biosynthetic pathway [40] (Additional file 3 and Additional file 5). On the other hand, agriGO annotated 127 different transcripts with increased expression in RIL165 with the “oxidation reduction” GO term. Of these, four are predicted orthologues of the rice cytochrome p450 genes; *CYP701A8* (GRMZM2G161472), *CYP71Z6* (GRMZM2G067591 and GRMZM2G122654) and *CYP993A* (GRMZM2G140448) which have been shown to play a role in oryzalexin, phytocassane and momilactone production, respectively [15, 17, 41] (Additional file 3 and Additional file 5).

**Fig. 4:**
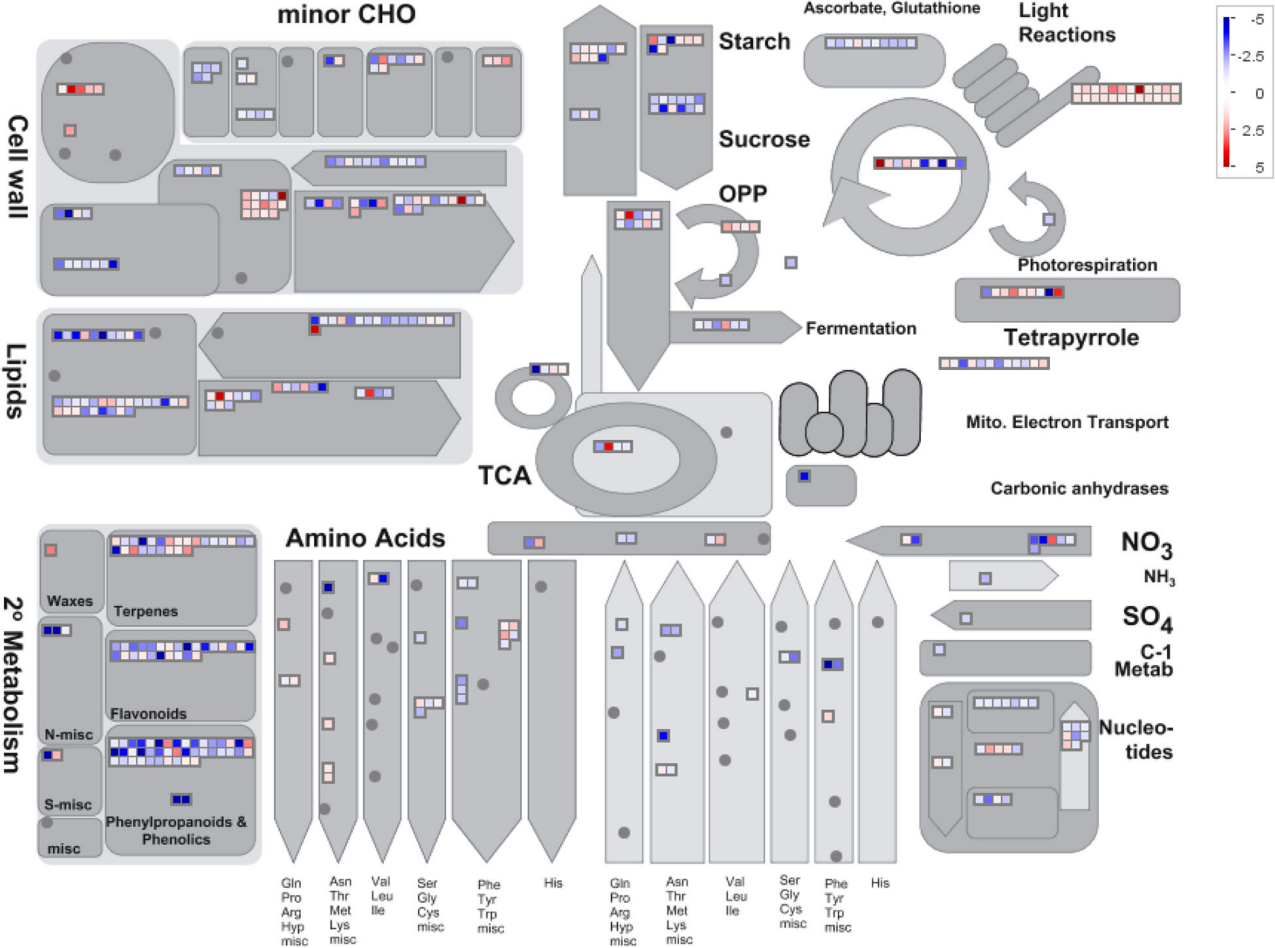
Overview of metabolic changes in RIL165 and RIL387, visualised by MapMan. Each entity within a pathway is depicted by a colour signal where red signifies genes with higher expression in RIL387 compared to RIL165 and blue signifies genes with higher expression in RIL165 compared to RIL387. The intensity of the colour indicates the level of expression [44]. Scale bar displays Log_2_fold changes. Genes binned to each pathway is available in Additional file 3

As an additional level of analysis, and to gain a clear understanding of the participation of metabolic pathways in defence responses to *C. zeina*, MapMan [42] was used to classify, or bin, all significant differentially expressed transcripts/enzymes with a Log_2_FC ≥ 1 or ≤ − 1 into metabolic pathways and processes (Fig. 4). An added advantage of MapMan ontology is that it is tailored specifically toward maize and Arabidopsis, whereas GO annotations are species-unspecific [43, 44]. Most differentially expressed genes were allocated into bins across primary and secondary metabolism, whereas only transcripts with higher expression in the resistant line, RIL387 (Log_2_FC ≥ 1) were placed in the bin “light reactions” corresponding to an enrichment of GO terms in the “photosynthesis” category (Fig. 3b; Fig. 4; Additional file 3). Other RIL387 transcripts were allocated to “wax” and “cell wall” bins, corresponding to an enrichment of GO terms in the “cellulose biosynthetic process” category (Fig. 3b; Fig. 4; Additional file 3). Transcripts allocated to the “cell wall” bin encode inter alia fasciclin-like arabinogalactan protein (GRMZM2G001514, GRMZM2G084812, GRMZM2G174799) and 1,3-β-D-glucan biosynthetic proteins (GRMZM2G465764, GRMZM2G471139, GRMZM2G084802) (Additional file 3).

Highly expressed transcripts from both RIL165 and RIL387 were allocated into the MapMan “secondary metabolism” bin, which also emerged in a parallel KEGG pathway analysis using the MADIBA tool [45] (Additional file 3; Additional file 6). This bin included sub-categories such as flavonoids, phenylpropanoids and phenolics, and terpenes. Classification of genes up-regualted in RIL165 onto these defense-associated secondary metabolism pathways corresponded to the enriched “response to biotic stimulus” GO term identified for RIL167 (Fig. 3). The sub-category “terpene secondary metabolism” was of interest as it included genes up-regulated in both RIL387 and RIL165. Maize accumulates diterpenoid kauralexins and sesquiterpenoid zealexins in response to a variety of abiotic and biotic stressors and these compounds have been shown to have antimicriobial activity [7, 22, 24, 25]. Several transcripts allocated to the terpene secondary metabolism bin have been shown to catalyze reactions leading to zealexins and kauralexins. We thus opted to investigate the role of maize phytoalexins in the responses of two maize lines to *C. zeina*, and followed the global transcriptome analysis with targeted phytoalexin profiling and gene expression analysis of phytoalexin biosynthetic genes.

### Identity of phytoalexins in maize in response to *C. zeina*

RNA-Seq data revealed the differential expression of several genes encoding enzymes with predicted function in terpenoid biosynthesis. The sesquiterpenoid zealexin biosynthetic gene *Tps6* (GRMZM2G127087) and the diterpenoid kauralexin biosynthetic gene, *ZmAn2* (GRMZM2G044481) had higher expression in RIL165 compared to RIL387 (Log_2_FC = −2.2 and −3.1 respectively; Additional file 3). Furthermore, two additional differentially expressed genes were identified from the RNA-Seq data as “*diterpene phytoalexins precursor biosynthesis”* genes based on their GO annotation, putative function and relatedness to rice orthologues as determined by the Plant Metabolic Network (PMN, www.plantcyc.org) [20]. The first “*diterpene phytoalexins precursor biosynthesis”* gene AC214360.3_FG001 (annotated as *ZmKSL2* [46]*;* Log_2_FC = −2.5) had a higher abundance of transcripts in RIL165 and possibly encodes a diterpene cyclase, *ent-pimara-8(14),15-diene synthase.* Based on phylogenetic analysis, this gene is predicted to be an orthologue of *OsKSL5* that encodes an enzyme which produces the metabolite *ent*-pimara-8(14),15-diene from *ent*-CDP (Fig. 1) [47, 48]. Thus AC214360.3_FG001 is a candidate kaurene synthase-like encoding gene. Interestingly, the second “*diterpene phytoalexins precursor biosynthetic*” gene, GRMZM2G068808 was significantly up-regulated in RIL387 (annotated as *ZmCPS3* by Schmelz and co-workers [44]*;* Log_2_FC = 1.5). Once again, based on phylogenetic analysis, this gene is a predicted orthologue of *OsCyc1,* a gene isolated from rice that encodes a *syn*-copalyl diphosphate synthase (*syn*-CPS) that converts GGPP into *syn*-CDP (Fig. 1). OsCyc1 is known to be involved in rice phytoalexin biosynthesis [49]. Thus, GRMZM2G068808 is a putative *syn*-CPS encoding gene.

We next investigated the induction of phytoalexin biosynthetic gene expression and phytoalexin accumulation in both RILs using a glass house time course analysis of maize leaves inoculated with *C. zeina*. Leaf material was harvested at three time points based on development of GLS disease symptoms: immediately after inoculation (0dpi, control), development of chlorotic spots (14 dpi) and development of grey leaf spot lesions (24 dpi for RIL165 and 28 dpi for RIL387). Disease symptoms did not progress in RIL387 to the same extent as RIL165, with leaves displaying only a few slightly elongated chlorotic lesions at the site of fungal inoculation after 4 weeks (28dpi; Additional file 7).

**Fig. 5:**
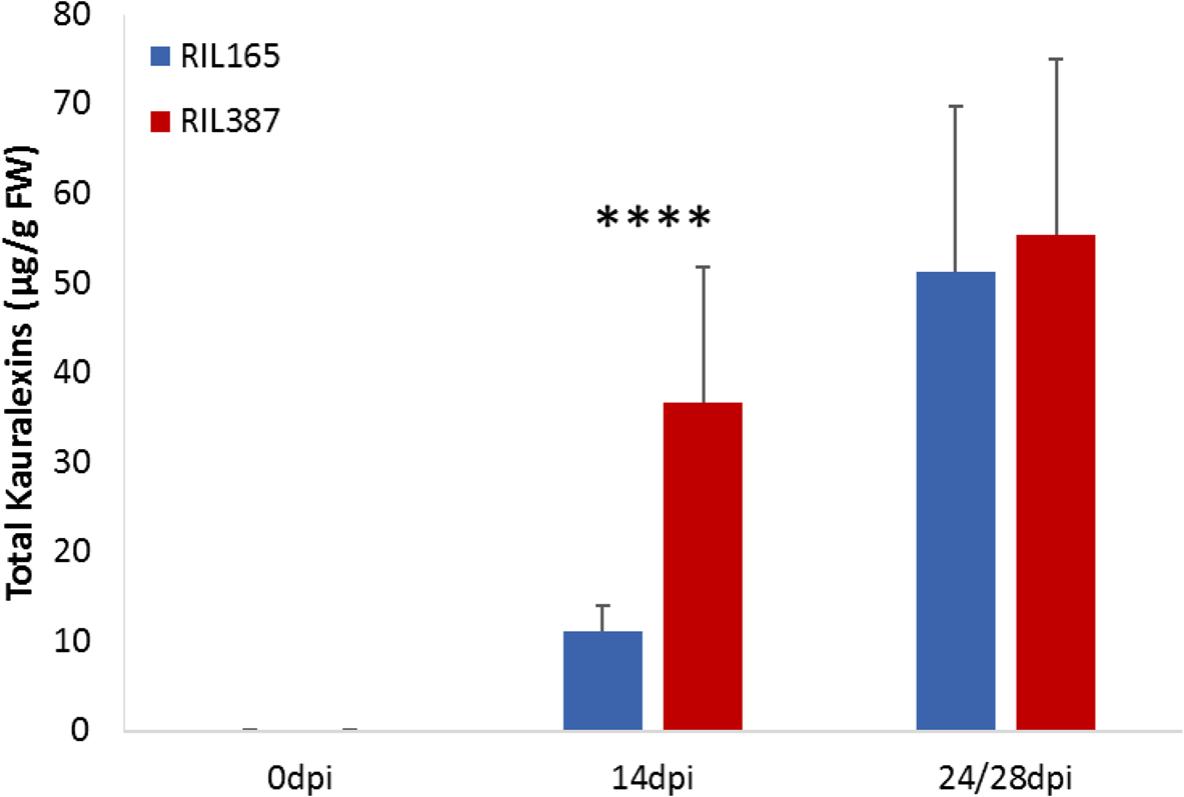
Total kauralexin accumulation in RIL165 and RIL387. Average levels (n = 3–5; ±SEM) of total kauralexin metabolites depicted on the primary y-axis in RIL165 and RIL387. Leaves were treated with a spore solution (3 × 10^5^ conidia/ml) and harvested at 0 days post inoculation (dpi), 14dpi and 24 or 28dpi (RIL165 and RIL387 respectively). Asterisks represent significant differences in expression levels between RIL165 and RIL387 (all ANOVAs, *P* < 0.0001; Tukey test corrections for multiple comparisons, *P* < 0.05)

Targeted terpenoid phytoalexin quantification was performed using gas chromatography/chemical ionization–mass spectrometry to determine if the differential expression of phytoalexin biosynthetic gene transcripts in the two lines could be correlated to phytoalexin accumulation. The analysis revealed that *C. zeina* inoculated leaves accumulated both families of phytoalexins (Fig. 5, Additional file 8); however, total zealexins were more abundant than total kauralexins in both genotypes. This is the second time that a predominance of zealexins have been reported in response to fungal infection [22]. No significant difference in total zealexin content was observed between the *C. zeina*-susceptible and *C. zeina* resistant RIL (Additional file 8). In contrast, there was a significant difference in the total kauralexins accumulating at 14dpi between RIL165 and RIL387 (*P* < 0.0001) (Fig. 5). Based on these observations, we were motivated to further investigate kauralexin accumulation.

### Differential accumulation of kauralexins in maize RILs with different levels of resistance to C. Zeina

**Fig. 6:**
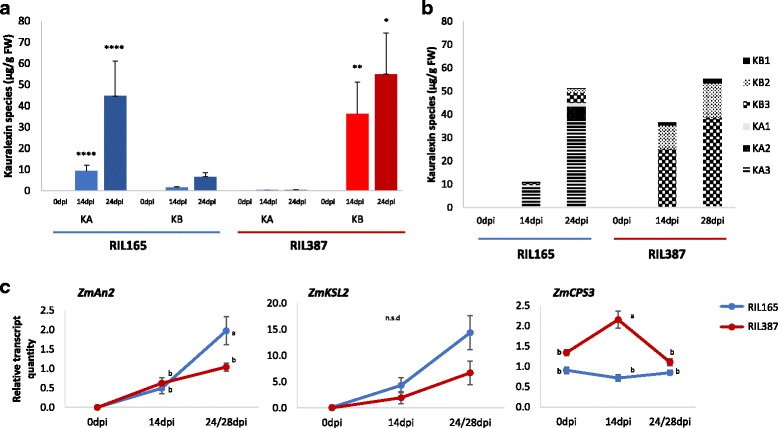
Kauralexin species accumulation in RIL165 and RIL387. **a** An abundance of kauralexin A compounds were measured in RIL165 leaf material as opposed to RIL387, where leaves contained majority kauralexin B compounds. Average levels (n = 3–5; ±SEM) of total kauralexin A and B metabolites in RIL165 and RIL387 depicted on the y-axis. Leaves were treated with a spore solution (3 × 10^5^ conidia/ml) and harvested at 0 days post inoculation (dpi), 14dpi and 24 or 28dpi (RIL165 and RIL387 respectively). **b** Distinct accumulation of kauralexin A3 and B3 compounds in RIL165 and RIL387, respectively. In RIL165, kauralexin A3 comprised 92% and 85% of the total kauralexin diterpenoids at 14dpi and 24dpi respectively, whereas in RIL387 70% of the total kauralexin diterpenoids consisted of kauralexin B3 at both 14dpi and 28dpi. **c** Average (n = 3–5; ±SEM) RT-qPCR relative transcript levels of genes encoding *ZmAn2, ZmKSL2* and *ZmCPS3*. Relative expression analysis was performed in qBase + v2.6 (BioGazelle, Zwijnaarde, Belgium), normalised against four reference genes. Lower case letter (**a**–**b**) and asterisks represent significant differences in expression levels between RIL165 and RIL387. All ANOVAs; **** P < 0.0001; ** *P* < 0.005; *P < 0.01. Tukey test corrections for multiple comparisons, P < 0.05

All six kauralexins species were detected in both RIL165 and RIL387. Interestingly, *C. zeina*-susceptible RIL165 accumulated primarily an excess of kauralexin A diterpenoids after *C. zeina* inoculation (Fig. 6a) and kauralexin A3 comprised 92% and 85% of the total kauralexin diterpenoids at 14dpi and 24dpi respectively (Fig. 6b). In contrast, *C. zeina*-resistant RIL387 showed a substantial accumulation of kauralexin B compounds (Fig. 6a), and 70% consisted of kauralexin B3 at both 14dpi and 28dpi (Fig. 6b).

We examined the expression of *ZmAn2, ZmCPS3* and *ZmKSL2* in response to *C. zeina* inoculation by quantitative RT-PCR (RT-qPCR) in order to determine if gene expression corresponded to kauralexin accumulation. *ZmAn2* expression was not detected in either *C. zeina*-resistant RIL387 or *C. zeina*-susceptible RIL165 at 0dpi, but transcripts accumulated at 14dpi and significantly increased in RIL165 from 14 to 24dpi after *C. zeina* inoculation (*p* ≤ 0.001) (Fig. 6c). Similarly, *C. zeina*-resistant RIL387 showed a comparable accumulation of *ZmAn2* transcripts at 14dpi but only a negligible increase at 28dpi (Fig. 6c). *ZmAn2* expression was significantly different between the lines at 24/28dpi and therefore verifies the RNA-Seq data (Log_2_FC = −3.1). *ZmKSL2* expression patterns mirrored that of *ZmAn2*, showing increased expression over time in both lines, although there was no significant difference at any point between the lines (Fig. 6c). *ZmAn2* and *ZmKSL2* expression was correlated to kauralexin A-type accumulation in *C. zeina*-susceptible RIL165 at 0dpi, 14dpi and 24dpi (Pearson correlation coefficients for both = 0.99) (Fig. 6c). *ZmAn2* and *ZmKSL2* transcript accumulation was also correlated with kauralexin B-type production in *C. zeina*-resistant RIL387 (Pearson correlation coefficients 0.99 and 0.91 respectively). However, despite similar *ZmAn2* and *ZmKSL2* expression at 14dpi in RIL165 and RIL387, the type of kauralexin species accumulated between the RILs were strinkingly dissimilar.

Interestingly, RIL387 had considerably higher levels of *ZmCPS3* at 14dpi than RIL165 (Fig. 6c; Anova, p ≤ 0.001)*.* Thereafter, *ZmCPS3* transcript levels were decreased at 28dpi in RIL387, comparable to the basal expression observed at 0dpi. There was no direct correlation between *ZmCPS3* expression and kauralexin accumulation in RIL387 over time (Pearson correlation coefficient − 0.03). RIL165 *ZmCPS3* transcript levels remained unvaried throughout the time course experiment (Fig. 6c).

## Discussion

The main purpose of this study was to investigate the molecular basis of quantitative resistance to *C. zeina* in maize by transcriptome sequencing and targeted metabolic profiling of two maize RILs at different ends of the resistance spectrum. The two lines subjected to RNA-Seq were part of a larger RIL population used to conduct quantitative trait loci mapping for GLS disease severity [50]. RIL387 carries seven resistance alleles for the eight QTL previously identified (Additional file 1), whereas RIL165 has only five resistance alleles.

The main observations from the study were firstly that the *C. zeina*-susceptible RIL165 had higher expression of many defence-related genes than RIL387. It is possible that the amplitude, timing and speed of transcriptional activation was insufficient to mount a successful defence response. Secondly, terpene biosynthetic genes including putative phytoalexin biosynthetic genes were up-regulated in both lines. Expression of kauralexin biosynthetic genes corresponded to accumulation of kauralexins, a group of diterpenoid phytalexins. Furthermore, the resistant RIL387 accumulated the B-series of kauralexins and in contrast the susceptible RIL165 produced predominantly the A-series of kauralexins.

### Transcriptome responses in the susceptible RIL165 after *C. zeina* infection

RIL165 carries susceptibility alleles for QTL6, QTL9b and QTL10 (Additional file 1). DE genes that lie within the 2-LOD interval of these QTL and were expressed higher in the susceptible RIL165 did not necessarily reveal susceptibility mechanisms or genes (Additional file 3). Instead the response in RIL165 was characterized by defence-related genes, which may represent downstream responses in this germplasm that are triggered too late. While RIL165 and RIL387 share four resistance alleles for QTL3a, QTL3b, QTL4 and QTL5, and RIL165 contains a further resistance allele for QTL9a, these appear to be insufficient to prevent disease development in RIL165. RIL165 may be defective in surveillance and pathogen detection mechanisms, which in the resistant RIL387 are conferred by the QTL alleles.

RNA-Seq expression data revealed that genes encoding chitinases, pathogenesis- related (PR) enzymes which digest the fungal cell wall, were strongly represented in *C. zeina* infected RIL165, contributing to the enriched GO term “chitin catabolism” (GO:0006559), and exhibiting various levels of expression in response to *C. zeina* (Additional file 3 and Additional file 5). As components of fungal cell walls, chitin oligosaccharides act as microbe- or pathogen- associated molecular patterns (MAMPs/PAMPs). Plants have developed extensive mechanisms to perceive chitin elicitors and respond by triggering innate defence responses [51]. For instance, studies have shown that chitin fragments induce up-regulation of enzymes participating in biosynthesis of terpenoid phytoalexins in rice [52–54] although chitosan did not induce phytoalexin accumulation in maize [55]. Genes representing other classes of pathogenesis-related (*PR*) genes were likewise induced upon infection with *C. zeina* in RIL165. These include genes encoding five Bowman–Birk serine proteinase inhibitors, four thaumatin-like proteins and four PR proteins (Additional file 3 and Additional file 5).

Jasmonate (JA), ethylene (Et) and salicylic acid (SA) signalling precedes the induction of genes encoding PR proteins such as chitinases [23, 56]. This led us to further interrogate the list of highly expressed genes in RIL165 for genes possibly involved in JA and SA biosynthesis and signalling. Genes encoding a putative lipoxygenase, allene oxide synthase, allene-oxide cyclase were identified (Additional file 3 and Additional file 5). These enzymes are predicted to participate in the conversion of linolenic acid to methyl-JA [57]. Besides JA, an Et biosynthetic gene, 1-aminocyclopropane-1-carboxylate oxidase (ACO), as well as several Et-responsive transcription factors were highly expressed in RIL165, suggesting that both JA and Et signalling takes place in a *C. zeina*-maize pathosystem, specifically in a susceptible genotype. No genes associated with salicylic acid (SA) biosynthesis or signalling were identified. This is in keeping with observations that defence responses to necrotrophic organisms are SA-independent and supports the hemibiotrophic character of *C. zeina* [58].

Several highly-expressed transcripts in RIL165 were categorised in the MapMan Flavonoid and MapMan phenylpropanoid and phenolics secondary metabolism bin (Fig. 4). Of these, six putative phenylalanine ammonia lyase (PAL) transcripts also contributed to the enriched GO term, “L-phenylalanine metabolism” (GO:0006032) in RIL165. The PAL enzyme is central to the phenylpropanoid pathway and converts L-phenylalanine into building blocks for phenolic metabolites such as lignans and flavonoids [59, 60], a class of phytoalexins in rice and oats [61–63]. Flavonoids such as 3-deoxyanthocyanidins have been shown to accumulate in a *Fusarium*-maize interaction [64]. A similar up-regulation of L-phenylalanine biosynthetic genes has been shown in wheat and barley in response to *F. graminearum*, aphids and *Puccinia hordei,* respectively [65–67]. In maize, infection by the biotroph *U. maydis* alters the expression of genes both upstream and downstream of L-phenylalanine, leading to an attenuated defence response [24]. Six genes encoding putative enzymes that catalyze reactions in the shikimate pathway were more abundant in RIL165, particularly the genes encoding predicted 3-dehydroquinate dehydratases, shikimate kinase, prephenate dehydratase, and aspartate aminotransferase. In plants, the shikimate pathway yields the building blocks, tyrosine and L-phenylalanine, for the biosynthesis of flavonoid compounds [60]. In addition, eight genes encoding enzymes that are predicted to partake in the flavonoid pathway - 4-coumarate coenzyme-A ligase, chalcone synthase, chalcone-flavonone isomerase, flavanone 3-hydroxylase, dihydroflavonol-4-reductase, and leucoanthocyanidin dioxygenase - were correspondingly increased in RIL165 compared to RIL387 (Additional file 3 and Additional file 5). Our results are similar to a previous study where extensive induction of flavonoid biosynthesis took place in maize kernels in response to *F. verticillioides* [35]*.* Maize flavonoids such as naringenin chalcone, apigenin and genkwanin have been shown to exhibit dose-dependent reduction of *C. graminicola* growth in vitro [68]*.*

Terpenoid phytoalexins are another family of critical antimicrobial compounds which accumulate in rice and maize in response to both biotic and abiotic stressors [7, 22, 25] and act to inhibit fungal growth. Four terpenoid biosynthetic genes were found to be highly expressed in the *C. zeina*-susceptible RIL165; *ent*-*copalyl diphosphate synthase* (*ZmAn2*), *ent-pimara-8(14),15-diene synthase* (*ZmKSL2*), and *Tps6*, the gene encoding homologous (S)-β-macrocarpene synthase. It is known that several microsomal cytochrome p450 monooxygenases have functional roles in phytoalexin production in rice [41, 69, 70] as well as phytoanticipin production in the grasses [40]. AgriGO also annotated 53 transcripts with increased expression in RIL165 as having monooxygenase activity (Additional file 3 and and Additional file 5). Of these, four are orthologues of the rice cytochrome p450 genes; *CYP701A8, CYP71Z6,* and *CYP99A3* which have been shown to play a role in oryzalexin, phytocassane and momilactone production, respectively [15, 17, 41]. Furthermore, a fifth cytochrome p450 gene, *CYP71Z18* (GRMZM2G067591) has recently been implicated in the oxidisation of β-macrocarpene to form zealexin A1 [71] and was also highly expressed in RIL165 (Log_2_FC = −1.7; Additional file 3 and Additional file 5). In summary, *C. zeina*-susceptible RIL165 appears to be mounting a multi-layered defence response against *C. zeina* involving PR proteins, JA synthesis, flavonoids and terpenoid phytoalexins, presumably in a final attempt at controlling fungal growth.

### Transcriptome responses in the resistant maize RIL387

Contrary to RIL165, RIL387 carries resistance alleles for QTL6, QTL9b and QTL10 and a susceptibility allele for QTL9a (Additional file 1). Many differentially expressed genes (*n* = 151, Log_2_FC ≥ 1 or ≤ − 1) were identified in this dataset that lie within the 2-LOD interval of these QTL and therefore represent candidate genes for the QTL if a transcriptional basis for the QTL can be identified. However, the transcriptomic response in RIL387 was complex and revealed the involvement of several processes including the production of secondary metabolites (Figs 3 and 4).

A reduction in photosynthetic potential from growth after biotic stress has been regarded as the underlying cost of launching an active defence response, as energy requirements are channelled from primary metabolism toward producing defence related molecules [72, 73]. Genes partaking in photosynthesis and chlorophyll biosynthesis are then usually globally down regulated [72, 73]. However, this was not what we observed in the resistant RIL387 transcriptome, which was enriched for GO terms such as “photosynthesis” (GO:0015979), and “pigment metabolic process” (GO:0042440). This may be the result of higher expression of photosynthesis genes in RIL387 or reduction in the amount of photosynthetically active tissue in RIL165 due to the extensive leaf blighting from *C.zeina* infection (Fig. 2).

Interestingly, GO terms such as “microtubule-based processes” (GO:0007018), “cellulose biosynthetic process” (GO:0030244) and “response to hormone stimulus” (GO:0009725) in RIL387 inoculated with *C. zeina* were overrepresented (Additional file 3 and Additional file 5). Plants use both structural and metabolic defence mechanisms to protect themselves against infection [74]. There is evidence that plant cells undergo large morphological alterations in reaction to pathogen attack in order to reinforce the cell wall, such as the response of Arabidopsis to *Verticillium dahliae* toxins [75]. Changes include cytoskeletal rearrangements facilitated by microtubule-based processes [76, 77] and it has been demonstrated that biosynthetic pathway genes of several compounds associated with cell-wall formation are up-regulated in response to corn borer feeding [78]. Furthermore, it has been hypothesised that quantitative disease resistance is conditioned by genes that regulate morphological changes [79]. Here we report that eight genes assigned the GO term “cellulose synthase” and therefore predicted to be involved in cell wall biosynthesis were highly expressed in RIL387 (Additional file 3 and Additional file 5). Although not assigned a GO term, GRMZM2GO57590 is also a putative cellulose synthase and maps to QTL9b (Additional file 3). Although physical changes in the cell wall structure were not confirmed in this study, cellulose synthases were up-regualted in barley against *Fusarium* head blight and in sorghum in response to methyl-jasmonate [80, 81]. In rice, genes involved in cell wall synthesis and modification are regulated by auxin [82]. Auxins play an integral role in plant growth and development, including transcription, signal transduction, metabolism, and transport [82]. In our study, seven auxin response transcription factors (ARF; ARF1, 3, 4, 6, 7, 15 and 20) were highly expressed in RIL387 - contributing to the enriched GO term “response to hormone stimulus” (GO:0009725) - presumably regulating gene expression in response to auxin (Additional file 3 and Additional file 5). In addition, two putative auxin response proteins that have not been assigned an ARF GO term (GRMZM2G074427 and GRMZM2G441325) were identified that map to QTL6 (Additional file 3). Auxins have also been implicated in the plant stress response but display complex behaviour in plant-pathogen interactions. For instance, some studies report that inhibition of auxin responses have led to disease resistance against bacteria, fungi and viruses [83, 84].

The RIL387 transcriptome did not show strong evidence for the role of the JA pathway in resistance to *C. zeina*. This is in contrast to our previous work on co-expression analysis of the RIL population, which showed expression of the orthologue of the JA receptor, COI-1, to be highly correlated with resistance [21]*.* In the current study, COI-1 (GRMZM2G151536) and a paralogue (GRMZM2G079112) were not differentially expressed between RIL387 and RIL165, indicating that their correlation with resistance in the larger RIL population may be due to QTL that are invariant between these two RILs.

Several secondary metabolite biosynthetic genes were differentially expressed between RIL165 and RIL387, and although these genes did not map to one of the *C. zeina* disease severity QTL with the resistance allele present in RIL367 only (QTL6, QTL9b and QTL10; Additional file 3), their differential expression indicated diversity between the two lines. Three terpenoid genes where expressed at a higher level in *C. zeina*-susceptible RIL165, and transcripts from a fourth gene, *ZmCPS3*, were more abundant in the *C. zeina*-resistant RIL387 than in RIL165 (Additional file 3). Many genes (*n* = 22), functionally annotated as having monooxygenase activity by agriGO, were highly expressed in RIL387 (Additional file 3 and Additional file 5), and therefore are potential phytoalexin or phytoanticipin biosynthetic genes. In the list of 22 highly expressed monooxygenases in RIL387, three benzoxazinoid (BX) biosynthetic genes, *Bx3, Bx4 and Bx5*, were identified. These genes encode enzymes which catalyze three successive reactions in the BX pathway and are clustered on chromosome 4 [40]. BXs are secondary metabolic compounds, with 2,4-dihydroxy-7-methoxy-1,4-benzoxazin-3-one (DIMBOA) being the major class in maize. Normally BXs are stockpiled and stored in the vacuole in an inactivate state [9, 85]. They are only activated and released when the plant is challenged by a pathogen, in order to combat the penetrating organism [9, 85]. A previous study has also reported de novo production of an anabolic product of DIMBOA-glc, 2-hydroxy-4,7-dimethoxy-1,4-benzoxazin-3-one-Glc (HDMBOA-Glc) [25].

DIMBOA is known to confer resistance to several maize pests including *Ostrinia nubilalis* and maize plant louse (*Rhophalosiphum maydis*) [86, 87]. DIMBOA-glc also co-accumulated with kauralexins in maize stems in response to herbivory [88] and DIMBOA-glc as well as the anabolite HDMBOA-glc increased in response to *Stagonospora nodorum* in combination with serotonin, a wheat phytoalexin [89]. There are also documented reports of DIMBOA-facilitated resistance to northern and southern corn leaf blight (fungal infections caused by *Helminthosporium turcicum* and *Bipolaris maydis* respectively) as well as *U. maydis* [28, 86, 90]. Ahmad et al. (2011) proposed that BXs (DIMBOA, DIMBOA-glc and HMDBOA-glc) have an additional role in penetration resistance, independent of tissue damage. During early infestation by *Setosphaeria turtica*, the authors observed a substantial impediment of *S. turtica* colonisation, coinciding with enhanced apoplastic BX deposition but prior to any host tissue damage [91].

To further investigate the participation of BXs in the response to *C. zeina*, we performed expression analysis of *Bx3, Bx4, Bx5* as well as two additional BX biosynthetic genes, *Bx1* and *Bx9,* and a gene excoding a β-glucosidase in RIL165 and RIL387 glass house material infected with *C. zeina* (Additional file 9). A clear pattern of up- or down-regulation was not observed across all genes, and although we did not test for benzoxizanoid accumulation, the role of DIMBOA-glu and HDMBOA-Glu in the resistance response to *C. zeina* remains inconclusive at this stage.

### Differential accumulation of zealexin and kauralexin compounds in RILs with contrasting phenotypes

Taken together, RNA-Seq data suggests that the susceptible RIL165 attempts to ward off *C. zeina* infection using an extensive arsenal of defence mechanisms. However, all efforts are ultimately overcome and the plant develops severe disease lesions. In comparison, the resistant RIL387 displays few immature lesions despite being exposed to the same degree of fungus, implying that RIL387 deployed an enhanced defensive barrier to *C. zeina,* be it structural or chemical. Allardyce et al. (2013) reported that secondary metabolite biosynthetic genes were up-regulated within 6 h after maize roots were inoculated with *P. cinnamomi*, but that expression subsequently decreased to basal levels after 24 h [23]. Similarly, other studies in maize stems have shown an initial sudden escalation in gene expression followed by a decline or plateau over time [7, 25]. Furthermore, it has been shown that induction of defence-related genes by *Colletotrichum graminicola* is organ specific and also occur later in leaves than in roots [92]. Therefore, it might be that RIL387 responds to *C. zeina* infection by expressing a different suite of genes compared to RIL165 that provide both a physical (through modification of the cell walls) and a chemical barrier.

We noted that both RIL387 and RIL165 had differential expression of terpenoid biosynthetic genes that provide the building blocks for phytoalexin production. Phytoalexins have been proposed to provide resistance to *C. zeina* infection [93], and therefore we investigated the induction of phytoalexin biosynthetic gene expression and phytoalexin accumulation in both genotypes using a glass house time course analysis of maize leaves inoculated with *C. zeina*. Both families of maize phytoalexins accumulated in response to *C. zeina*, albeit, zealexins were more abundantly elicited than kauralexins in both genotypes. This observation is in contrast to earlier reports where kauralexins are generally elicited to higher levels than zealexins [22, 94]. Furthermore, the abundance of zealexins in response to *C. zeina* infection is curious considering that a similar hemibiotrophic specialized maize pathogen, *C. graminicola,* was a poor elicitor of zealexin production in maize stems [25].

*C. zeina*-susceptible RIL165 accumulated significantly higher levels of the kauralexin A series diterpenoids than the levels observed for RIL387 (Fig. 5a; ANOVA *p* ≤ 0.05). However, development of C. zeina symptoms in RIL165 continued despite the accumulation of kauralexin A metabolites. In contrast, *C. zeina*-resistant RIL387 elicited substantially higher levels of kauralexin B series compounds than the levels observed in RIL165 (Fig. 5a; ANOVA p ≤ 0.05), and symptoms in RIL387 were limited to small chlorotic lesions, even after 46dpi (Additional file 7). Kauralexin A3 and B3 have previously been shown to be the most abundant kauralexins produced in response to insect and fungal attack [7] and displayed different levels of potency against different pathogens. For example, application of low doses of metabolite, such as 10 μg/mL kauralexin A3 did not reduce *Rhizopus microsporus* growth whereas the same dose of kauralexin B3 diminished *R. microsporus* growth by 30% [7]. On the other hand, both kauralexin A3 and B3 could delay the disease progression of the maize specialist, *C. graminicola* by 50–60% at 10 μg/mL [7] and kauralexin B3 was also able to reduce *F. verticillioides* growth by 30% [95]. Accordingly, kauralexin B3 appears to have direct anti-fungal activity at low doses, suggesting that kauralexin B3 might be an active component of *C. zeina* growth inhibition in RIL387.

Several studies have shown induction of *ZmAn2* expression in response to *F. graminearum* and *O. nubilalis*, followed by a significant accumulation of several kauralexin compounds [7, 25, 88]. However, in this context, *ZmAn2* expression does not explain the divergent accumulation of kauralexin A and B in RIL165 and RIL387 respectively (Fig. 5a). Furthermore, despite similar *ZmAn2* expression at 14dpi in RIL165 and RIL387, kauralexin accumulation was almost four-fold higher in RIL387 than RIL165 (Fig. 5), the majority of which consisted of kauralexin B metabolites (Fig. 5b). It has been proposed that the kauralexin A and B series are generated through the action of two distinct kaurene synthases [19].

To explain the differential accumulation of A and B series of kauralexin compounds, we investigated the putative diterpenoid biosynthetic gene that was highly expressed in the RNA-Seq dataset; *ent-pimara-8(14),15-diene synthase* (*ZmKSL2*; AC214360.3_FG001). *ZmKSL2* expression emulated the expression pattern observed for *ZmAn2* and was similar between the two lines, albeit lower in RIL387 (Fig. 5b). We have previously noted that *ZmAn2* and *ZmKSL2* are co-expressed and are correlated to lesion development in 100 RILs inoculated with *C. zeina* [21]. In rice, *OsKSL5* produces *ent*-pimara-8(14),15-diene from *ent*-CDP, a secondary metabolite that has not yet been implicated as a phytoalexin. We speculate that though ZmKSL2 has been predicted to act in the “*diterpene phytoalexins precursor biosynthetic*” pathway (Plant Metabolic Network (PMN, www.plantcyc.org) to yield *ent*-pimara-8(14),15-diene, it is possible that ZmKSL2 is acting downstream of *ent*-CPS in the kauralexin biosynthetic pathway. However, functional analysis is required to substantiate the role of *ZmKSL2*.

Interestingly, four highly expressed genes in the RIL387 transcriptome data encode enzymes that function in the cytosolic mevalonate (MVA) pathway pathway that yield the terpenoid building block, farnesyl diphosphate (FPP). These were *3-hydroxy-3-methylglutaryl-synthase, 3-hydroxy-3-methylglutaryl-coenzyme A reductase* (two paralogues) and *mevalonate diphosphate decarboxylase* (Additional file 3; GRMZM2G393337; GRMZM2G058095, GRMZM2G087207, and GRMZM2G070351). Terpenoid building blocks - geranyl diphosphate (GPP), FPP and geranylgeranyl diphosphate (GGPP) - are derived from either the methylerythritol phosphate (MEP) or the MVA pathway [96]. Production of diterpenoids via GGPP in rice are expected to arise in plastids through the MEP pathway, whereas sesquiterpene production via FPP generally occur in the cytoplasm via the MVA pathway [96, 97]. Terpenoid biosynthesis is catalyzed by terpene synthases (TPS), a diverse class of enzymes that convert GPP, FPP and GGPP to an assortment of monoterpenoid, sesquiterpenoid, and diterpenoid skeletons respectively [98].

Contrary to RIL387, RIL165 transcriptome data showed an excess of specific transcripts that yield enzymes of both the MVA and the MEP pathway (Additional file 3; GRMZM2G102550, GRMZM5G830250, GRMZM2G058404, GRMZM2G147721, GRMZM2G493395, GRMZM2G163809, GRMZM2G098569). Specifically, a major regulatory enzyme, 1-deoxy-D-xylulose 5-phosphate synthase (first enzyme in the MEP pathway, GRMZM2G058404) and geranyl-geranyl diphosphate synthase (GRMZM2G493395), the ultimate enzyme that produces GGPP (Additional file 3) were identified. Lanubile et al., (2014) previously reported enhanced constitutive expression of primarily MEP pathway genes in a *F. verticillioides -*resistant maize line as well as enhanced induction after infection [35]. Although up-regulation of MVA pathway intermediates may link to accumulation of zealexins in both lines, there is no clear link between MEP pathway intermediates and kauralexin accumulation in our data. Regulation of the MVA and MEP pathway in plants is complex, and it has been shown in *Arabidopsis* that individual pathway transcripts accumulate differentially depending on different organs or developmental stages [97].

Notably, RIL387 had considerably higher levels of *ZmCPS3* (GRMZM2G068808) at 14dpi than RIL165. In rice, GGPP is converted to two stereochemically differentiated isomers, *ent*- and *syn*-CDP through cyclization catalyzed by two separate copalyl diphosphate synthases (CPS), *ent*-CPS and *syn*-CPS [10]. *ZmCPS3* is an ortholog of *OsCPS4*, encoding *syn*-CPS in rice. Rice kaurene synthase-like enzymes then produce phytocassanes A-E and oryzalexins A-F from *ent*-CDP but momilactones A and B, and oryzalexin S from *syn*-CDP [8, 13, 14]. Currently, kauralexins A and B are known to be derived through the action of *ent*-CPS only and *syn*-CPS derived phytoalexins have not yet been described in maize. It is unclear whether the observed intensification in expression of *ZmCPS3* in the resistant line leads to the production of novel phytoalexins, sufficiently potent to inhibit the progression of *C. zeina* infection, and highlights the need for a global metabolite screening.

## Conclusions

We investigated the defence response of a *C. zeina*-susceptible and a *C. zeina* resistant maize inbred line through transcriptome sequencing. These lines harbor different alleles at four GLS disease severity QTLs [21, 50]. We found that the *C. zeina*-susceptible RIL165 accumulates transcripts encoding proteins partaking in both primary and secondary metabolism as the plant defended itself against the fungal infection. Notably, changes in flavonoid and terpenoid biosynthetic genes, plant hormone signalling genes and genes encoding PR proteins were observed. The *C. zeina*-resistant RIL387 responded by accruing transcripts involved in cell wall modification, including auxin signalling genes. We confirm the accumulation of zealexins and kauralexins in both the resistant and susceptible maize line in response to *C. zeina*. In conjunction, *ZmAn2* as well as *ZmCPS3* was shown to be induced by *C. zeina* and differentially expressed between RIL165 and RIL387. Diversity in kauralexin accrual is described in this study. The kauralexin A and B series of compounds were substantially more abundant in the susceptible and resistant line respectively. A recent report showed that the B series of kauralexins are induced by drought and salt stress [22], while both kauralexins A and B accumulate equally after fungal elicitation [7], advocating for specific and discriminate roles for phytoalexins in response to different stressors. We propose that future endeavours should include global metabolite screening compelled by the discovery that *ZmCPS3* is induced by *C. zeina* in the resistant line and could produce novel terpenoid phytoalexins.

## Methods

### Plant material and RNA isolation

Two subtropical white dent inbred lines CML444 and SC Malawi were previously crossed to yield a recombinant inbred line population (RIL, F7:S6) [50, 99]. A field trial of this population under high GLS disease pressure was previously conducted at Baynesfield Estate, an experimental site in Kwazulu-Natal Province, South Africa in 2008/2009 [50]. Leaf material for RNA-Seq were collected from three biological replicates each of two lines at VT/R1 stage of development. These two lines were on opposite sides of the disease severity spectrum; a *C. zeina*-susceptible line (RIL165; disease severity score 7.5) and a *C. zeina*-resistant line (RIL387; disease severity score 1.5). The amount of *C. zeina* DNA in an aliquot of the same leaf samples used for RNA-Seq was quantified using qPCR as previously described [31].

*C. zeina*-susceptible RIL165 and *C. zeina*-resistant RIL387 seeds were planted in a greenhouse in 30 cm wide pots filled with a sand/coir mix. The temperature in the greenhouse was maintained at an average of 21 +/− 5 °C. The day length was 16 h. A HOBO® data logger was used to measure temperature and humidity in greenhouse. The pots were positioned on a turning table and coupled to a drip irrigation system which allowed plants to receive a total of 1000 ml water during two 5 min sessions. Eleven days after planting the following fertilizers were applied to the 5000-l tank: 3.2 kg OMNICAL™ calcium nitrate; 500 ml K-Max liquid potassium fertilizer and 5 kg HYDROGRO water soluble hydroponic fertilizer mix. At 36 days after planting (dap), misters were switched on 4 times a day for an hour to maintain a 100% humidified environment.

Once the plants reached V8-V10 stage (approximately 57 dap), each fully emerged leaf was marked with permanent black marker pen to designate an area approximately 10cm^2^ in the middle of the leaf blade. *C. zeina* culture CMW 25463 [31] was sporulated whilst grown on V8 medium and incubated in the dark. A hockey stick spreader was used to dislodge the conidia in a solution of 0.01% Tween 20. The spore solution (3 × 10^5^ conidia/ml) was painted onto both surfaces of all leaves with a small paint brush. The humidity was increased to 60% to maintain an optimal growth environment for *C. zeina*. Leaf pieces were sampled at three time points corresponding to three different disease stages for both RIL165 and RIL387 for DNA, RNA and metabolite extraction. These were: immediately after inoculation (0 dpi), at appearance of *chlorotic spots* (14 dpi) and finally once lesions were *fully developed* (24 dpi for RIL165) and *starting to elongate* (28 dpi for RIL387).

Total RNA from three biological replicates were extracted using QIAzol (Qiagen, Hilden, Germany) according to the manufacturer’s instructions, followed by RNase-free DNase (Qiagen, Hilden, Germany) treatment. Thereafter the samples were purified using the RNeasy Mini Kit (Qiagen, Hilden, Germany) as per the manufacturer’s instructions.

### RNA-Seq and data analysis

RNA library construction and sequencing were performed at Beijing Genomics Institute (Shenzhen, China) in accordance with manufacturer’s instructions. Maize leaf mRNA from the field trial was used to prepare 50 bp short-insert libraries using the TruSeq RNA Sample Prep Kit and sequencing was performed on a HiSeq™ 2000 system (Illumina Inc., San Diego, USA). Raw data was analyzed using a local instance of Galaxy, a web-based tool kit for bioinformatics analysis (http://galaxyproject.org/). Additional file 2 details the analysis pipeline which was used. Firstly, read quality was evaluated by the fastQC application v0.11.2. Illumina 1.5 encoded quality scores (Q) were converted to Sanger scale (phred) using FASTQ Groomer Galaxy v1.0.4 [100]. Thereafter, sequence reads for each biological replicate were mapped to v2 of the B73 reference genome (5b.60 annotation; sequence obtained from Phytozome v9.1) [101], using TopHat v2.0.9 (http://ccb.jhu.edu/software/tophat/index.shtml) [102], by implementing Bowtie2 v1.0.0 [103]. Intron length was specified as between 5 to 60,000 bp, mean inner distance between mate pairs defined as 100 bp and up to two mismatches allowed per 25 bp kmer. TopHat was instructed to apply a coverage search as a source of evidence for intron/exon boundaries. All other parameters were used with default values. Cufflinks v2.0.2 (http://cole-trapnell-lab.github.io/cufflinks/) [33] was used to calculate transcript abundance which is reported as fragments per kilobase pair of exon model per million fragments mapped (FPKM). The B73 reference v2 5b.60 filtered gene set (www.phytozome.org/maize) was supplied as a reference annotation and provided to enable the implementation of bias detection and correction. To improve calculation of differential expression for less abundant genes and transcripts, quartile normalization was applied. Default settings were maintained for all other parameters. Six assemblies consisting of three resistant and three susceptible biological replicate samples were generated by Cufflinks and merged with the reference annotation into a single .gtf file using Cuffmerge [33]. Differential expression analysis was conducted on the merged file using Cuffdiff [33, 34] with a False Discovery Rate (FDR) threshold set to 0.05. The subsequent list of differentially expressed (DE) genes was further filtered to reflect genes with a log_2_fold change (Log_2_FC) ≥1 or ≤ − 1 for downstream analysis.

### Data deposition

Transcriptome sequence data generated in this study was deposited in the National Center for Biotechnology Information (NCBI) Gene Expression Omnibus (GEO) collection, accession number GSE99005.

### Gene ontology enrichment and pathway analysis

DE genes were annotated using Blast2GO [104] and gene ontology enrichment analysis was carried out using agriGO v1.2 (http://bioinfo.cau.edu.cn/agriGO) [36]. Singular enrichment analysis (SEA) was performed using a hypergeometric test, Hochberg FDR adjustment method parameters, a significance level of 0.05, and a minimum number of five mapped entries using the complete set of gene ontology terms. MapMan [42, 44] and MADIBA (MicroArray Data Interface for Biological Annotation) [45] were utilised for biochemical pathway analysis.

### Quantitative real-time PCR (RT-qPCR)

A starting concentration of 500–2000 ng RNA was used to synthesize cDNA in duplicate using the High Capacity cDNA Synthesis Kit (Life Technologies) as per manufacturer’s protocol. Cycling was performed on the DNA Engine Tetrad 2 Peltier thermal cycler (Biorad, Hercules, California, United States) as per manufacturers protocol.

When not obtained from literature cited, primers targeting the GOI were designed using a combination of PerlPrimer v1.1.21 [105], Primer3Plus (http://www.bioinformatics.nl/cgi-bin/primer3plus/primer3plus.cgi/ [106] and Primer Express® v2.0 (Life Technologies, Carlsbad, California, United States). Standard, optimal RT-qPCR primer restrictions were adhered to for internal quality control. The primers spanned an intron/exon boundary and were specific to the 3’UTR where possible. Basic Local Alignment Search Tool (BLAST) compared the primer sequences against the maize genome (http://plants.ensembl.org/Tools/Blast?db=core) courtesy of Ensembl Plants (http://plants.ensembl.org/index.html) to assist in avoiding mis-priming to non-specific targets. Primer information is maintained in Additional file 10. Names of genes are abbreviated as per the Enzyme Commission Synonyms from Plant Metabolic Network (PMN) on www.plantcyc.org, July 2015.

Expression analysis was performed on the ABI 7900HT Fast Real Time PCR (Life Technologies, Carlsbad, California, United States) instrument using Power SYBR® Green PCR Master Mix (Life Technologies, Carlsbad, California, United States). Reactions contained 100 ng cDNA and 100–200 nM of primer.

Relative expression analysis was performed using qBase + v2.6 (BioGazelle, Zwijnaarde, Belgium) [107, 108]. Four efficiency corrected reference genes were used for target normalization as per the generalized qBase quantification model [107, 108]; viz. DNA directed RNA polymerase (GRMZM2G034326) [109], sr-like RNA binding protein gene (GRMZM2G127729), dag protein gene (GRMZM2G451729), and eukaryotic translation initiation factor 4e-2 gene (GRMZM2G445905).

One-way analysis of variance (ANOVA, with Tukey-Kramer post-test) followed by an unpaired t-test (with false discovery rate *p*-value adjustment) was performed in qBase + v2.6 (BioGazelle, Zwijnaarde, Belgium) [107, 108]. Data were log_10_ transformed prior to statistical analysis. Transcripts were considered differentially expressed if the adjusted p-value <0.05.

### Maize metabolite extraction and quantification

Leaf tissue (100–300 mg) from V8-V10 maize lines, RIL165 and RIL387 grown in a glass house and artificially inoculated with *C. zeina* was ground to a fine powder in liquid nitrogen and phytoalexins extracted according to a previously described protocol [7, 25]. The metabolite content of each sample was then analyzed using gas chromatography/chemical ionization – mass spectrometry using an established method [110, 111].

## Additional files

Additional file 1: Grey leaf spot (GLS) severity quantitative trait loci (QTLs) identified for the CML444 X SC Malawi maize recombinant inbred line population identified in the Baynesfield trial. List of disease severity QTL identified in RIL population [21, 50] and corresponding alleles in RIL165 and RIL387 (XLSX 11 kb)

Additional file 2: RNA-Seq data analysis pipeline. Read quality was evaluated by the fastQC application v0.11.2. Illumina 1.5 encoded quality scores (Q) were converted to Sanger scale (phred) using FASTQ Groomer Galaxy v1.0.4. Thereafter, sequence reads were mapped to v2 of the B73 reference genome (5b.60 annotation; sequence obtained from Phytozome v9.1), using TopHat v2.0.9 (http://ccb.jhu.edu/software/tophat/index.shtml/), by implementing Bowtie2 v1.0.0. Cufflinks v2.0.2 (http://cole-trapnell-lab.github.io/cufflinks//) was used to calculate transcript abundance, reported as fragments per kilobase pair of exon model per million fragments mapped (FPKM). Transcript assemblies were merged with the reference annotation into a single .gtf file using Cuffmerge. Differential expression analysis was conducted on the merged file using Cuffdiff with a False Discovery Rate (FDR) threshold set to 0.05 (PPTX 133 kb)

Additional file 3: Expression levels, protein annotation and GO annotation of differentially expressed genes. Statistically significant differentially expressed genes between the two genotypes are reported. The Log_2_FC derived from the comparisons of the fragments per kilobase of transcript per million fragments mapped (FPKM) expression values of RIL165 vs RIL387 is depicted. For each gene, the putative annotation of the protein according to the RefSeq database (provided by the National Center for Biotechnology Information (NCBI), www.ncbi.nlm.nih.gov/refseq/), Uniprot (Universal Protein Resource; www.uniprot.org) and GenBank sequence database (provided by the NCBI, www.ncbi.nlm.nih.gov/genbank/) is described. Gene ontology terms mapped to each gene by AgriGo are included, in addition to the chromosomal positions of the genes in v2 of the B73 reference genome. Where the DE gene overlapped with the genomic position of a disease severity QTL [21, 50] this was indicated (XLSX 564 kb)

Additional file 4: Comparison of RNA-Seq and RT-qPCR expression analyses of genes between RIL165 and RIL387. Expression profiles of **(a)** *ent-copalyl diphosphate synthase 2* (GRMZM2G044481), **(b)** *syn-copalyl diphosphate synthase* (GRMZM2G068808), **(c)** *Terpene synthase 6* (GRMZM2G127087_T03), **(d)** *β-glucosidase1* (GRMZM2G031660), **(e)** *Bx3* (GRMZM2G167549), **(f)** *Bx5* (GRMZM2G063756), **(g)** *Bx8* (GRMZM2G085054), and **(h)** *Bx9* (GRMZM2G161335) is depicted (PPTX 183 kb)

Additional file 5: Significant enriched GO terms and associated genes responsive to C. zeina infection in a susceptible (RIL165) maize line. Gene ontology enrichment analysis was carried out using agriGO v1.2 of statistically significant differentially expressed genes with a Log_2_FC > 1 or <−1. Singular enrichment analysis (SEA) was performed using a hypergeometric test, Hochberg FDR adjustment method parameters, a significance level of 0.05, and a minimum number of five mapped entries using the complete set of gene ontology terms (XLSX 399 kb)

Additional file 6: Overview of pathways where differentially expressed genes participate as reported by MADIBA. Up-regulated gene products were mapped onto metabolic pathways using the KEGG representation. The number of enzymes in each pathway is portrayed for both RIL165 and RIL387 (PPTX 961 kb)

Additional file 7: Photographs depicting GLS disease progression in RIL165 and RIL387 greenhouse material inoculated with C. zeina. Material was harvested at three time points based on development of GLS disease symptoms: immediately after inoculation (0dpi, control), development of chlorotic spots (14 dpi) and development of grey leaf spot lesions (24 dpi for RIL165 and 28 dpi for RIL387) (PPTX 1912 kb)

Additional file 8: Zealexin defences are induced in response to *C. zeina.* Leaves were treated with a spore solution (3 × 10^5^ conidia/ml) and harvested at 0 days post inoculation (dpi), 14dpi and 24 or 28dpi (RIL165 and RIL387 respectively). The metabolite content of each sample was analysed using gas chromatography/chemical ionization – mass spectrometry. Zealexins were quantified based on the internal standard ^13^C_18_-linolenic acid and presented in ng/μg FW. Average levels of total zealexin metabolites depicted for RIL165 and RIL387 (*n* = 3–5; ±SEM) (PPTX 89 kb)

Additional file 9: Biosynthesis of benzoxazinoids in maize. The biosynthetic pathway of DIMBOA is depicted as per [112]. The expression profiles of DIMBOA biosynthetic genes in glass house leaf material is presented (DOCX 142 kb)

Additional file 10: Primer sequences and descriptive information of genes studied (DOCX 23 kb)

## Abbreviations

ABA: abscisic acid
ACT: *Actin*
BLAST: basic local alignment search tool
bp: base pair
BTUB: B-tubelin
BX: benzoxazinoids
CPS: copalyl diphosphate synthase
Ct: cycle threshold
DAG: dag protein gene
DAP: days after planting
DE: differentially expressed
DIMBOA: 2,4-dihydroxy-7-methoxy-1,4-benzoxazin-3-one
DNA: deoxyribonucleic acid
dNTP: deoxynucleotide triphosphates
dpi: days post inoculation
*ent*-CPS: *ent*-copalyl diphosphate synthase
Et: ethylene
ETIF: eukaryotic translation initiation factor 4e-2 gene
FDR: false discovery rate
FHB: Fusarium head blight
FPKM: fragments per kilobase of exon per million fragments mapped
FPP: farnesyl diphosphate
GGPP: geranylgeranyl diphosphate
GLS: grey leaf spot
GO: gene ontology
GOI: genes of interest
GPP: geranyl diphosphate
JA: jasmonic acid
Log_2_FC: Logarithm (base 2) fold change
LUG: *Leunig*
MADIBA: microarray data interface for biological annotation
MEP: methyl-D-erythritol 4-phosphate
MEV: mevalonate
ng: nanogram
nM: nano molar
PAL: l-phenylalanine ammonia lyase
PCR: polymerase chain reaction
pg: picogram
PR: pathogenesis related
qPCR: quantitative polymerase chain reaction
R genes: resistance genes
RIL: recombinant inbred line
RNA: ribonucleic acid
RNase: ribonuclease
RPOL: DNA directed RNA polymerase
RT-qPCR: real-time quantitative polymerase chain reaction
SA: salicylic acid
SEA: singular enrichment analysis
SRL: sr-like RNA binding protein gene
Tm: melting temperature
TPS: terpene synthase
