# Supplementary material for: RNA-Seq analysis of resistant and susceptible sub-tropical maize lines reveals a role for kauralexins in resistance to grey leaf spot disease, caused by Cercospora zeina
Source: BMC Plant Biol. 2017 Nov 13;17:197. doi: 10.1186/s12870-017-1137-9 (PMC5683525; doi:10.1186/s12870-017-1137-9)
Supplement: Supplementary file 2 — RNA-Seq data analysis pipeline. Read quality was evaluated by the fastQC application v0.11.2. Illumina 1.5 encoded quality scores (Q) were converted to Sanger scale (phred) using FASTQ Groomer Galaxy v1.0.4. Thereafter, sequence reads were mapped to v2 of the B73 reference genome (5b.60 annotation; sequence obtained from Phytozome v9.1), using TopHat v2.0.9 (http://ccb.jhu.edu/software/tophat/index.shtml/), by implementing Bowtie2 v1.0.0. Cufflinks v2.0.2 (http://cole-trapnell-lab.github.io/cufflinks//) was used to calculate transcript abundance, reported as fragments per kilobase pair of exon model per million fragments mapped (FPKM). Transcript assemblies were merged with the reference annotation into a single .gtf file using Cuffmerge. Differential expression analysis was conducted on the merged file using Cuffdiff with a False Discovery Rate (FDR) threshold set to 0.05 (PPTX 133 kb) [file 12870_2017_1137_MOESM2_ESM.pptx]

## Slide 1
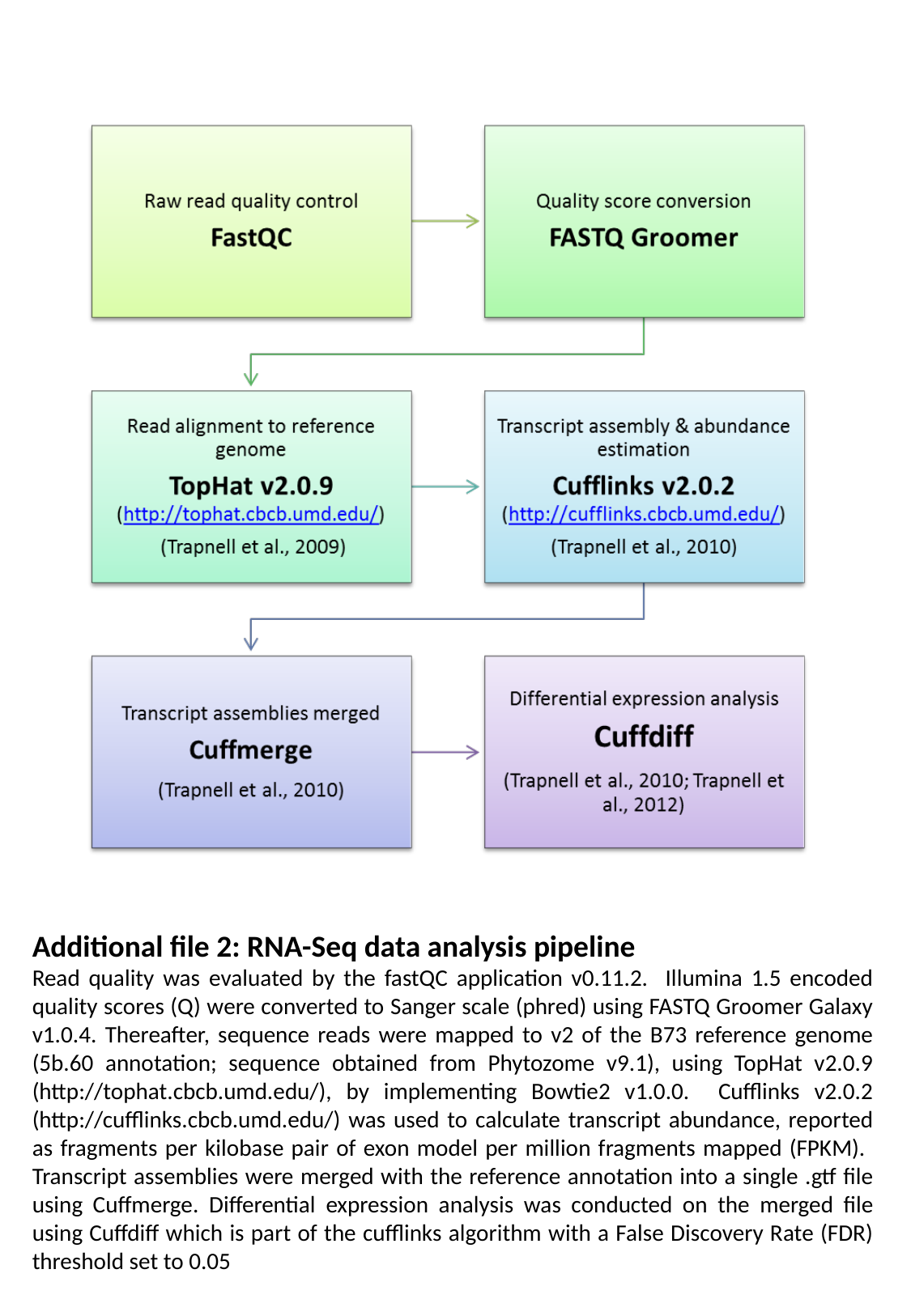

Additional file 2: RNA-Seq data analysis pipeline
Read quality was evaluated by the fastQC application v0.11.2. Illumina 1.5 encoded quality scores (Q) were converted to Sanger scale (phred) using FASTQ Groomer Galaxy v1.0.4. Thereafter, sequence reads were mapped to v2 of the B73 reference genome (5b.60 annotation; sequence obtained from Phytozome v9.1), using TopHat v2.0.9 (http://tophat.cbcb.umd.edu/), by implementing Bowtie2 v1.0.0. Cufflinks v2.0.2 (http://cufflinks.cbcb.umd.edu/) was used to calculate transcript abundance, reported as fragments per kilobase pair of exon model per million fragments mapped (FPKM). Transcript assemblies were merged with the reference annotation into a single .gtf file using Cuffmerge. Differential expression analysis was conducted on the merged file using Cuffdiff which is part of the cufflinks algorithm with a False Discovery Rate (FDR) threshold set to 0.05
