# Supplementary material for: RNA-Seq analysis of resistant and susceptible sub-tropical maize lines reveals a role for kauralexins in resistance to grey leaf spot disease, caused by Cercospora zeina
Source: BMC Plant Biol. 2017 Nov 13;17:197. doi: 10.1186/s12870-017-1137-9 (PMC5683525; doi:10.1186/s12870-017-1137-9)
Supplement: Supplementary file 4 — Comparison of RNA-Seq and RT-qPCR expression analyses of genes between RIL165 and RIL387. Expression profiles of (a) ent-copalyl diphosphate synthase 2 (GRMZM2G044481), (b) syn-copalyl diphosphate synthase (GRMZM2G068808), (c) Terpene synthase 6 (GRMZM2G127087_T03), (d) β-glucosidase1 (GRMZM2G031660), (e) Bx3 (GRMZM2G167549), (f) Bx5 (GRMZM2G063756), (g) Bx8 (GRMZM2G085054), and (h) Bx9 (GRMZM2G161335) is depicted (PPTX 183 kb) [file 12870_2017_1137_MOESM4_ESM.pptx]

## Slide 1
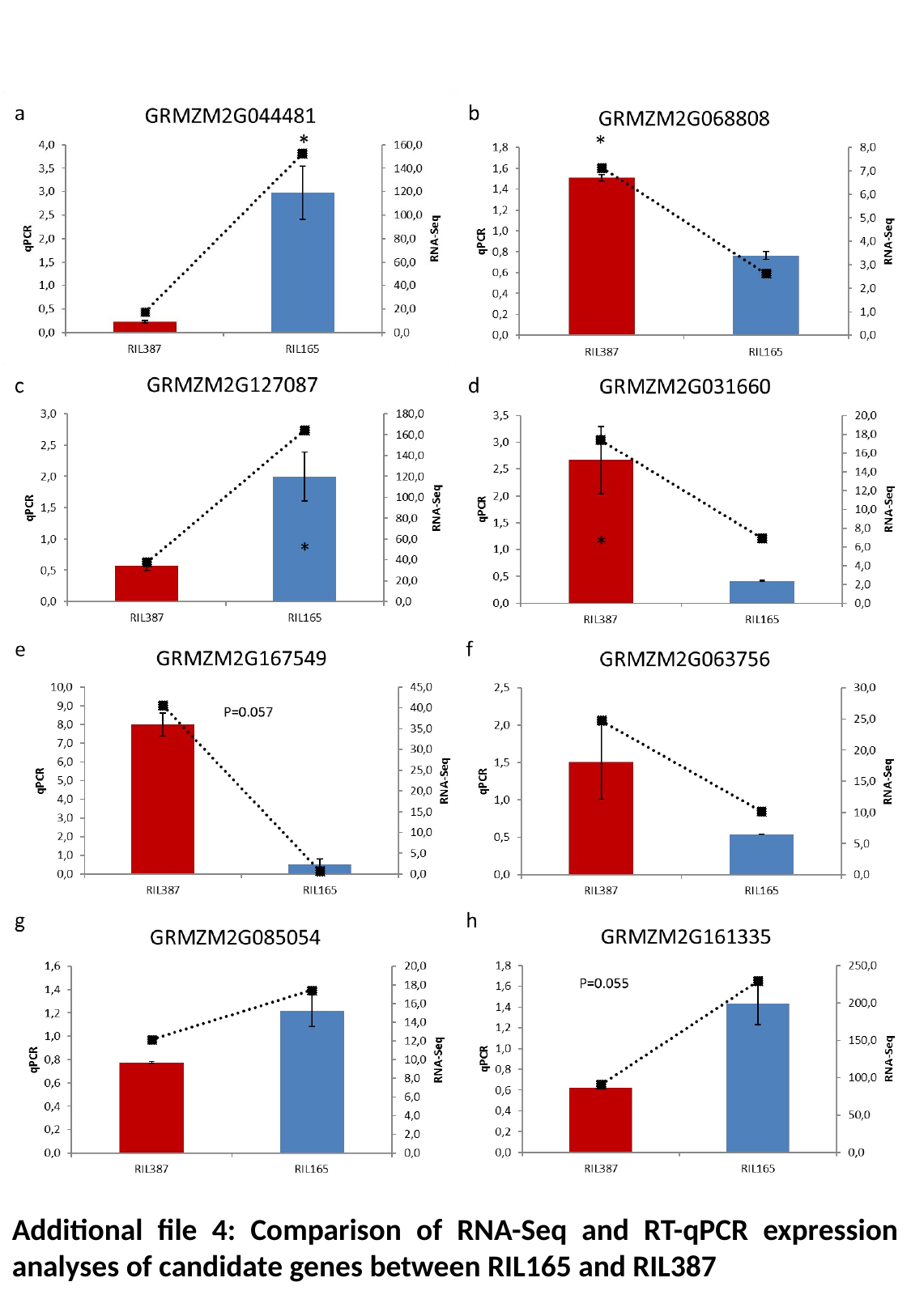

Additional file 4: Comparison of RNA-Seq and RT-qPCR expression analyses of candidate genes between RIL165 and RIL387

## Slide 2
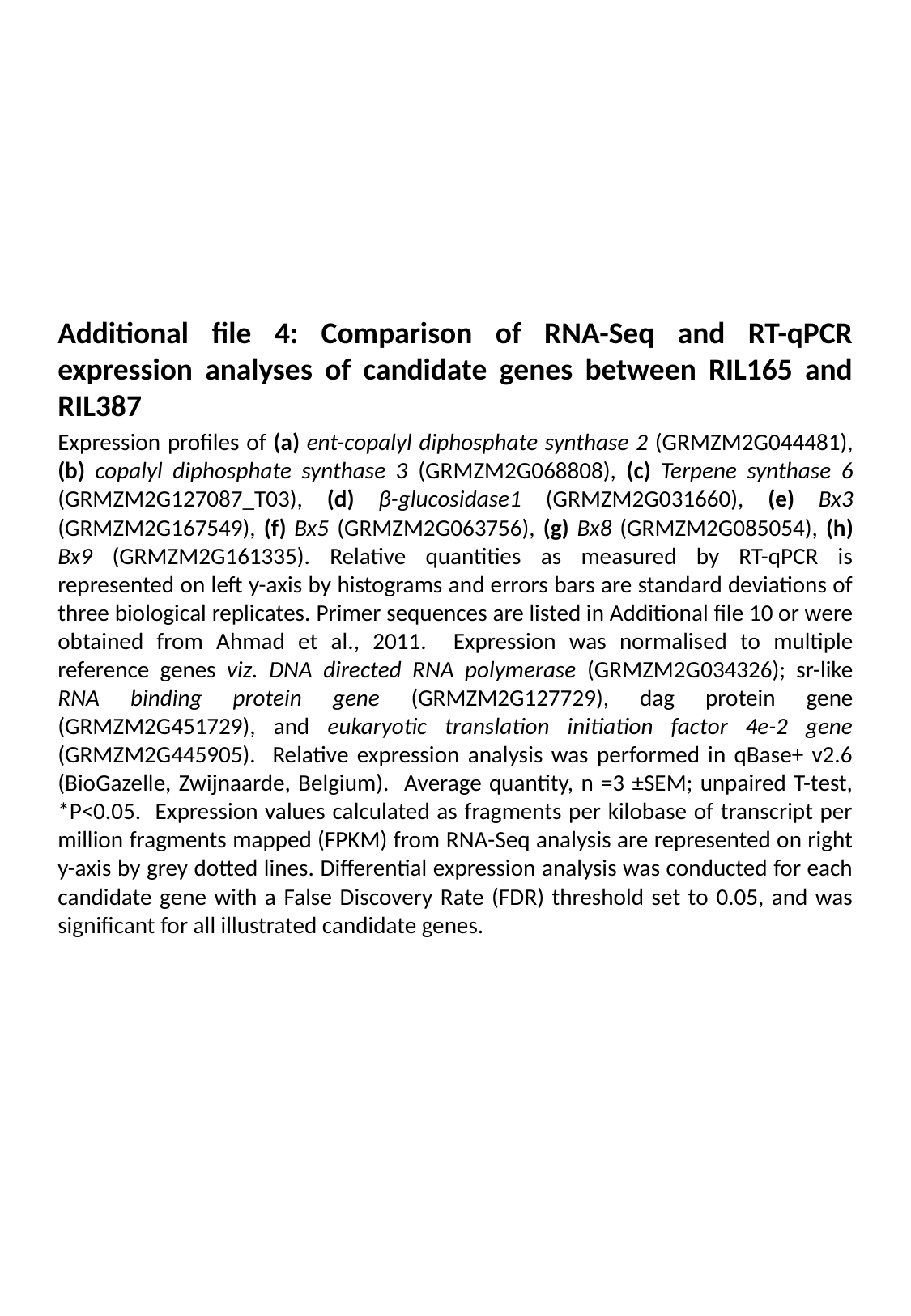

Additional file 4: Comparison of RNA-Seq and RT-qPCR expression analyses of candidate genes between RIL165 and RIL387
Expression profiles of (a) ent-copalyl diphosphate synthase 2 (GRMZM2G044481), (b) copalyl diphosphate synthase 3 (GRMZM2G068808), (c) Terpene synthase 6 (GRMZM2G127087_T03), (d) β-glucosidase1 (GRMZM2G031660), (e) Bx3 (GRMZM2G167549), (f) Bx5 (GRMZM2G063756), (g) Bx8 (GRMZM2G085054), (h) Bx9 (GRMZM2G161335). Relative quantities as measured by RT-qPCR is represented on left y-axis by histograms and errors bars are standard deviations of three biological replicates. Primer sequences are listed in Additional file 10 or were obtained from Ahmad et al., 2011. Expression was normalised to multiple reference genes viz. DNA directed RNA polymerase (GRMZM2G034326); sr-like RNA binding protein gene (GRMZM2G127729), dag protein gene (GRMZM2G451729), and eukaryotic translation initiation factor 4e-2 gene (GRMZM2G445905). Relative expression analysis was performed in qBase+ v2.6 (BioGazelle, Zwijnaarde, Belgium). Average quantity, n =3 ±SEM; unpaired T-test, *P<0.05. Expression values calculated as fragments per kilobase of transcript per million fragments mapped (FPKM) from RNA-Seq analysis are represented on right y-axis by grey dotted lines. Differential expression analysis was conducted for each candidate gene with a False Discovery Rate (FDR) threshold set to 0.05, and was significant for all illustrated candidate genes.
