# Supplementary material for: RNA-Seq analysis of resistant and susceptible sub-tropical maize lines reveals a role for kauralexins in resistance to grey leaf spot disease, caused by Cercospora zeina
Source: BMC Plant Biol. 2017 Nov 13;17:197. doi: 10.1186/s12870-017-1137-9 (PMC5683525; doi:10.1186/s12870-017-1137-9)
Supplement: Supplementary file 6 — Overview of pathways where differentially expressed genes participate as reported by MADIBA. Up-regulated gene products were mapped onto metabolic pathways using the KEGG representation. The number of enzymes in each pathway is portrayed for both RIL165 and RIL387 (PPTX 961 kb) [file 12870_2017_1137_MOESM6_ESM.pptx]

## Slide 1
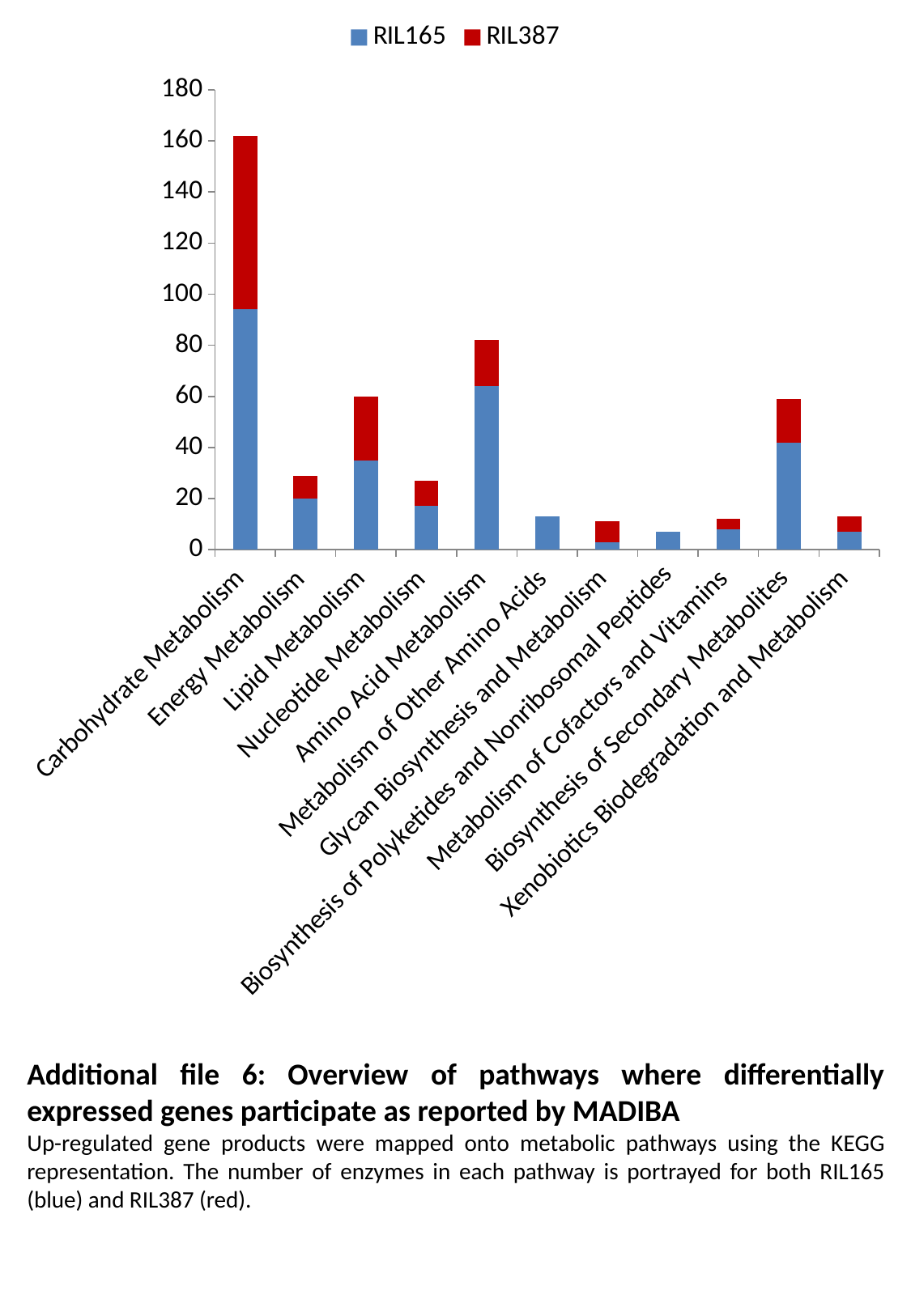

### Chart
| Category | RIL165 | RIL387 |
|---|---|---|
| Carbohydrate Metabolism  | 94.0 | 68.0 |
| Energy Metabolism  | 20.0 | 9.0 |
| Lipid Metabolism  | 35.0 | 25.0 |
| Nucleotide Metabolism  | 17.0 | 10.0 |
| Amino Acid Metabolism  | 64.0 | 18.0 |
| Metabolism of Other Amino Acids  | 13.0 | 0.0 |
| Glycan Biosynthesis and Metabolism  | 3.0 | 8.0 |
| Biosynthesis of Polyketides and Nonribosomal Peptides | 7.0 | 0.0 |
| Metabolism of Cofactors and Vitamins  | 8.0 | 4.0 |
| Biosynthesis of Secondary Metabolites  | 42.0 | 17.0 |
| Xenobiotics Biodegradation and Metabolism  | 7.0 | 6.0 |Additional file 6: Overview of pathways where differentially expressed genes participate as reported by MADIBA
Up-regulated gene products were mapped onto metabolic pathways using the KEGG representation. The number of enzymes in each pathway is portrayed for both RIL165 (blue) and RIL387 (red).
