# Supplementary material for: RNA-Seq analysis of resistant and susceptible sub-tropical maize lines reveals a role for kauralexins in resistance to grey leaf spot disease, caused by Cercospora zeina
Source: BMC Plant Biol. 2017 Nov 13;17:197. doi: 10.1186/s12870-017-1137-9 (PMC5683525; doi:10.1186/s12870-017-1137-9)
Supplement: Supplementary file 7 — Photographs depicting GLS disease progression in RIL165 and RIL387 greenhouse material inoculated with C. zeina. Material was harvested at three time points based on development of GLS disease symptoms: immediately after inoculation (0dpi, control), development of chlorotic spots (14 dpi) and development of grey leaf spot lesions (24 dpi for RIL165 and 28 dpi for RIL387) (PPTX 1912 kb) [file 12870_2017_1137_MOESM7_ESM.pptx]

## Slide 1
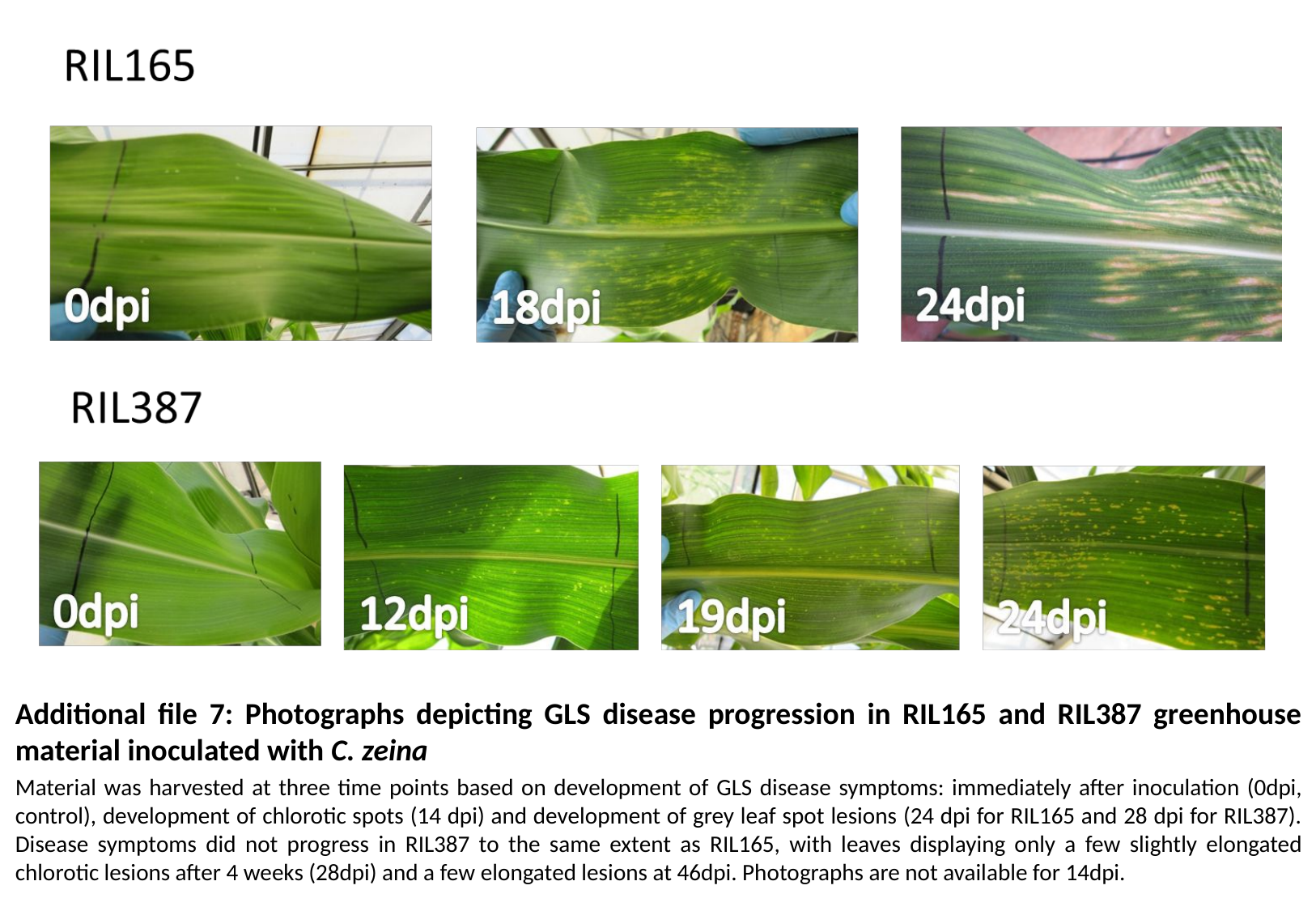

Additional file 7: Photographs depicting GLS disease progression in RIL165 and RIL387 greenhouse material inoculated with C. zeina
Material was harvested at three time points based on development of GLS disease symptoms: immediately after inoculation (0dpi, control), development of chlorotic spots (14 dpi) and development of grey leaf spot lesions (24 dpi for RIL165 and 28 dpi for RIL387). Disease symptoms did not progress in RIL387 to the same extent as RIL165, with leaves displaying only a few slightly elongated chlorotic lesions after 4 weeks (28dpi) and a few elongated lesions at 46dpi. Photographs are not available for 14dpi.
