# Supplementary material for: RNA-Seq analysis of resistant and susceptible sub-tropical maize lines reveals a role for kauralexins in resistance to grey leaf spot disease, caused by Cercospora zeina
Source: BMC Plant Biol. 2017 Nov 13;17:197. doi: 10.1186/s12870-017-1137-9 (PMC5683525; doi:10.1186/s12870-017-1137-9)
Supplement: Supplementary file 8 — Zealexin defences are induced in response to C. zeina. Leaves were treated with a spore solution (3 × 10^5^ conidia/ml) and harvested at 0 days post inoculation (dpi), 14dpi and 24 or 28dpi (RIL165 and RIL387 respectively). The metabolite content of each sample was analysed using gas chromatography/chemical ionization – mass spectrometry. Zealexins were quantified based on the internal standard 13C18-linolenic acid and presented in ng/μg FW. Average levels of total zealexin metabolites depicted for RIL165 and RIL387 (n = 3–5; ±SEM) (PPTX 89 kb) [file 12870_2017_1137_MOESM8_ESM.pptx]

## Slide 1
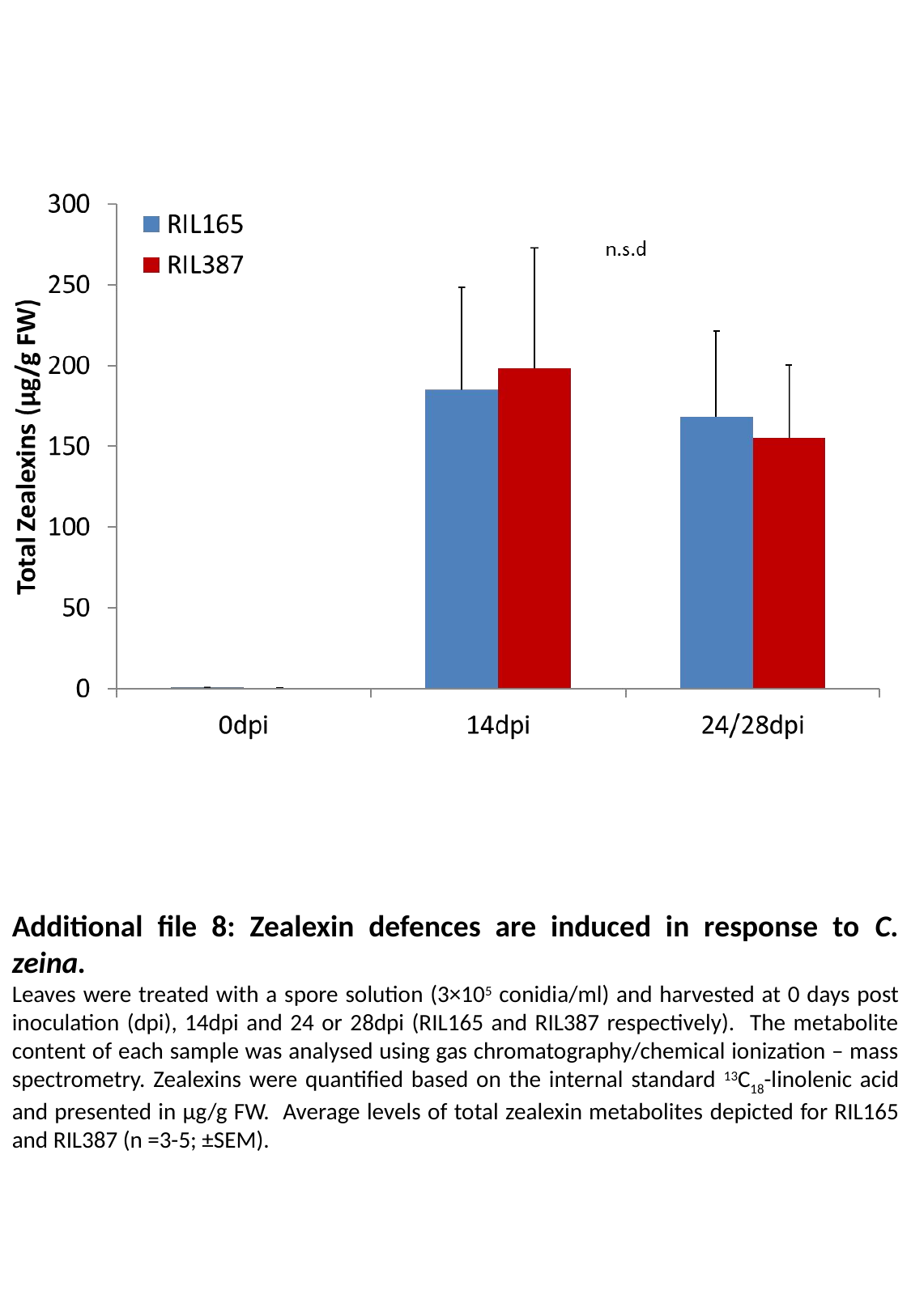

Additional file 8: Zealexin defences are induced in response to C. zeina.
Leaves were treated with a spore solution (3×10^5^ conidia/ml) and harvested at 0 days post inoculation (dpi), 14dpi and 24 or 28dpi (RIL165 and RIL387 respectively). The metabolite content of each sample was analysed using gas chromatography/chemical ionization – mass spectrometry. Zealexins were quantified based on the internal standard 13C18-linolenic acid and presented in µg/g FW. Average levels of total zealexin metabolites depicted for RIL165 and RIL387 (n =3-5; ±SEM).
