# Supplementary material for: RNA-Seq analysis of resistant and susceptible sub-tropical maize lines reveals a role for kauralexins in resistance to grey leaf spot disease, caused by Cercospora zeina
Source: BMC Plant Biol. 2017 Nov 13;17:197. doi: 10.1186/s12870-017-1137-9 (PMC5683525; doi:10.1186/s12870-017-1137-9)
Supplement: Supplementary file 9 — Biosynthesis of benzoxazinoids in maize. The biosynthetic pathway of DIMBOA is depicted as per [112]. The expression profiles of DIMBOA biosynthetic genes in glass house leaf material is presented (DOCX 142 kb) [file 12870_2017_1137_MOESM9_ESM.docx]

**
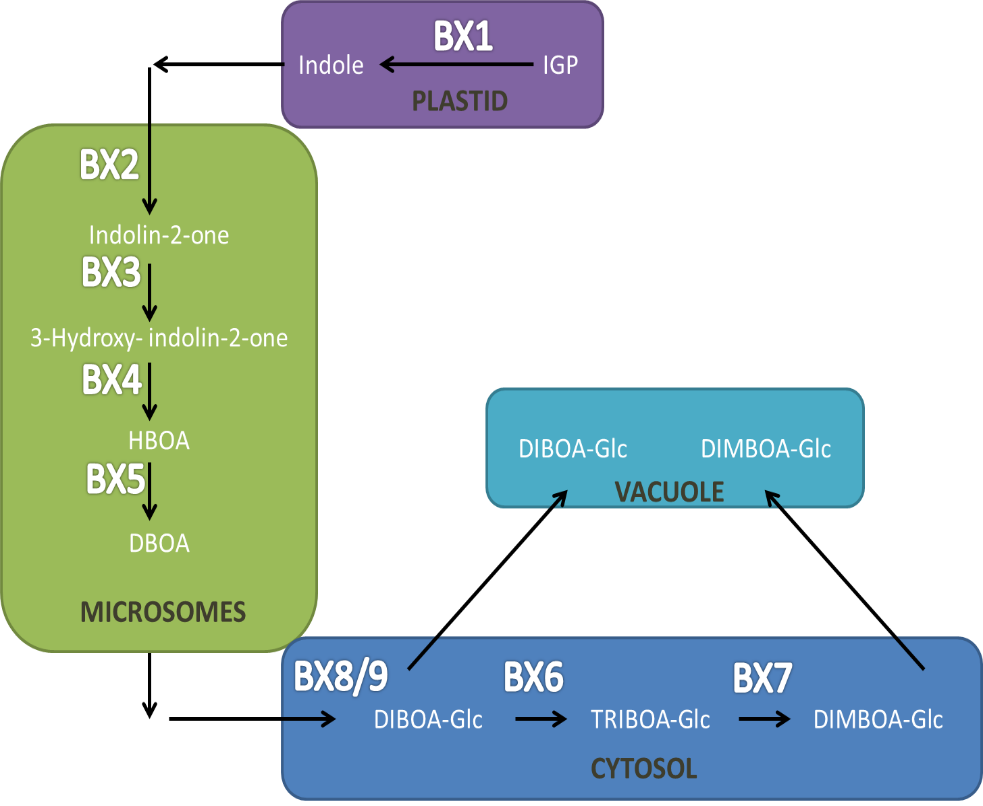
**

|  |  | 0dpi | | 14dpi | | 24/28dpi | |
| --- | --- | --- | --- | --- | --- | --- | --- |
|  |  | **Ave** | ***SEM*** | **Ave** | ***SEM*** | **Ave** | ***SEM*** |
| BX1 | **RIL165** | 5,48 | *1,39* | 4,59 | *2,75* | 1,68 | *0,37* |
|  | **RIL387** | 6,78 | *2,30* | 0,67 | *0,05* | 0,33 | *0,08* |
| BX3 | **RIL165** | 0,94 | *0,09* | 0,33 | *0,04* | 0,17 | *0,01* |
|  | **RIL387** | **11.17 *** | *0,82* | **4.13 *** | *1,02* | **2.51 *** | *0,47* |
| BX4 | **RIL165** | 1,81 | *0,82* | 0,6 | *0,10* | 2,19 | *0,64* |
|  | **RIL387** | 1,43 | *0,32* | 1,41 | *0,24* | 0,89 | *0,16* |
| BX5 | **RIL165** | 1,92 | *1,03* | 0,15 | *0,03* | 1,71 | *0,88* |
|  | **RIL387** | 6,25 | *1,09* | **2.45 *** | *0,39* | 1,66 | *0,42* |
| BX9 | **RIL165** | 1,78 | *0,02* | 1,1 | *0,03* | 0,58 | *0,14* |
|  | **RIL387** | 1,83 | *0,14* | 0,94 | *0,07* | **1.04 *** | *0,04* |

**Additional file 9: Biosynthesis of benzoxazinoids in maize**

**Figure:** The first committed step in the BX pathway is defined by the conversion of indole-3-glycerole phosphate (IGP) to indole by BX1 in the plastid (Frey et al., 1997). Thereafter, four successive oxygen atoms are introduced into the indole moiety within the microsomes by four distinct members of the CYP71 family of cytochrome P450 dependent monooxygenases (P450s); BX2 to BX5 (Frey et al., 1995). BX2 first catalyses the conversion of indole to indolin-2-one. Thereafter indolin-2-one is transformed to 3-hydroxy-indolin-2-one by BX3. Subsequently 2-hydroxy-2-1,4-benzoxazin-3-one (HBOA) is produced by BX4 and finally HBOA is converted to DIBOA after N-hydroxylation effected by BX5. At this point, DIBOA is glucosidated by two serial UDP-glucosyltransferases (UGTs) BX8 and BX9 into DIBOA-glucoside within the cytosol (von Rad et al., 2001). The conversion of DIBOA-glc to inactive DIMBOA-glc is mediated by the cytosolic dioxygenase, BX6 (Frey et al., 2003) and a methyltransferase, BX7 (Jonczyk et al., 2008). DIMBOA-glc accumulates in the vacuole as an inactive, but stable, glucoside to prevent auto-toxicity (Frey et al., 2009).

**Table:** Expression profiles of *Bx1*, (GRMZM2G085381), *Bx3* (GRMZM2G167549), *Bx4* (GRMZM2G172491), *Bx5* (GRMZM2G063756), and *Bx9* (GRMZM2G161335) in RIL165 compared to RIL387. Primer sequences obtained from Ahmad et al., 2011. Relative expression analysis was performed in qBase+ v2.6 (BioGazelle, Zwijnaarde, Belgium) and normalised to four reference genes *viz. DNA directed RNA polymerase* (GRMZM2G034326); *sr-like RNA binding protein* gene (GRMZM2G127729), *dag protein* gene (GRMZM2G451729), and *eukaryotic translation initiation factor* *4e-2* gene (GRMZM2G445905). Gene expression that was significantly different between the lines are indicated in **bold** font. Average quantity, n =3 ±SEM; unpaired T-test, *P<0.05. *Figure adapted from Frey M, Schullehner K, Dick R, Fiesselmann A, Gierl A: Benzoxazinoid biosynthesis, a model for evolution of secondary metabolic pathways in plants. Phytochemistry 2009,* ***70****:1645–1651.*
