# Supplementary material for: RNA-Seq analysis of resistant and susceptible sub-tropical maize lines reveals a role for kauralexins in resistance to grey leaf spot disease, caused by Cercospora zeina
Source: BMC Plant Biol. 2017 Nov 13;17:197. doi: 10.1186/s12870-017-1137-9 (PMC5683525; doi:10.1186/s12870-017-1137-9)
Supplement: Supplementary file 10 — Primer sequences and descriptive information of genes studied (DOCX 23 kb) [file 12870_2017_1137_MOESM10_ESM.docx]

**Additional file 10: Primer sequences and descriptive information of genes studied**

| **Reference or Target Gene**  **Name (in this study)** | **Reference** | **Reference**  **Gene ID** | **CornCyc/Uniprot** | **RefSeq** | **Chr** | **Gene Product** | **Primer Sequences (5'-3')** | **Product**  **size** |
| --- | --- | --- | --- | --- | --- | --- | --- | --- |
| *DAG* | Bridget Crampton, pers comm | GRMZM2G451729 |  |  | 2 | dag protein | F: CAACCATCAGCACCTGGAT | 120 |
|  |  |  |  |  |  |  | R: TGGGCTAGGATACTGACCAGA |  |
| *ETIF* | Bridget Crampton, pers comm | GRMZM2G445905 |  |  | 1 | eukaryotic translation  initiation factor 4e-2 | F: AGAAGGGATAAGCCAAGCAG | 104 |
|  |  |  |  |  |  |  | R: CCGATCTCAGCACAAAACTC |  |
| *RPOL* | [2] | GRMZM2G034326 |  |  | 5 | DNA directed RNA polymerase I, II, III | F: AGCCAAAACGCTAAAGTGGA | 175 |
|  |  |  |  |  |  |  | R: TAAGTGACGAGCAAGGCAAA |  |
| *SRL* | Bridget Crampton, pers comm | GRMZM2G127729 |  |  | 2 | nuclear sr-like RNA binding protein | F: ACACGCCATTGTTCGAGA | 117 |
|  |  |  |  |  |  |  | R: CAGGTTCGGGTGAACTTTG |  |
| *Tps6* | [3] | GRMZM2G127087_T03 | G-12279/ K7TGS7 | NM_001112480/ NP_001105950 | 10 | terpene synthase 6 | F: GAAATGCGACAAAGGGCTG | 398 |
|  |  |  |  |  |  |  | R: TCTTGAAGGCATCTCGTAGTA |  |
| *An2* | [4] | GRMZM2G044481 | G-14667/ Q672R4 | NM_001111787.1 | 1 | *ent*-copalyl diphosphate synthase 2 | F: TGTTCTTGTGAAGGCAGTTC | 236 |
|  |  |  |  |  |  |  | R: TCATTCGAGCTAAAAGCAGA |  |
| *CPS3* | This study | GRMZM2G068808 | GDQC-104441  /K7THR6 | XM_008664609.1 | 10 | copalyl diphosphate synthase | F: AGCCTTAGATGGATCGTTGAG | 159 |
|  |  |  |  |  |  |  | R: GAATGACAAACCTTTCTCGCAC |  |
| *KSL2* | This study | AC214360.3_FG001 | GDQC-5387/ B7ZYQ8 | DAA54948 | 3 | kaurene syntase-like 2 | F: ACTCATCTCCGCTCACGAAT | 88 |
|  |  |  |  |  |  |  | R: ACCGGGGAGTTGATCTTCTT |  |
